# Supplementary material for: Synthesis and Activity of Ionic Antioxidant-Functionalized PAMAMs and PPIs Dendrimers
Source: Polymers (Basel). 2022 Aug 27;14(17):3513. doi: 10.3390/polym14173513 (PMC9459880; doi:10.3390/polym14173513)
Supplement: Supplementary file 1 [file polymers-14-03513-s001.zip › polymers-1852228-supplementary.pdf]

Figure S1 : ^{1}H NMR of PPI-1-PhA in CD_3OD

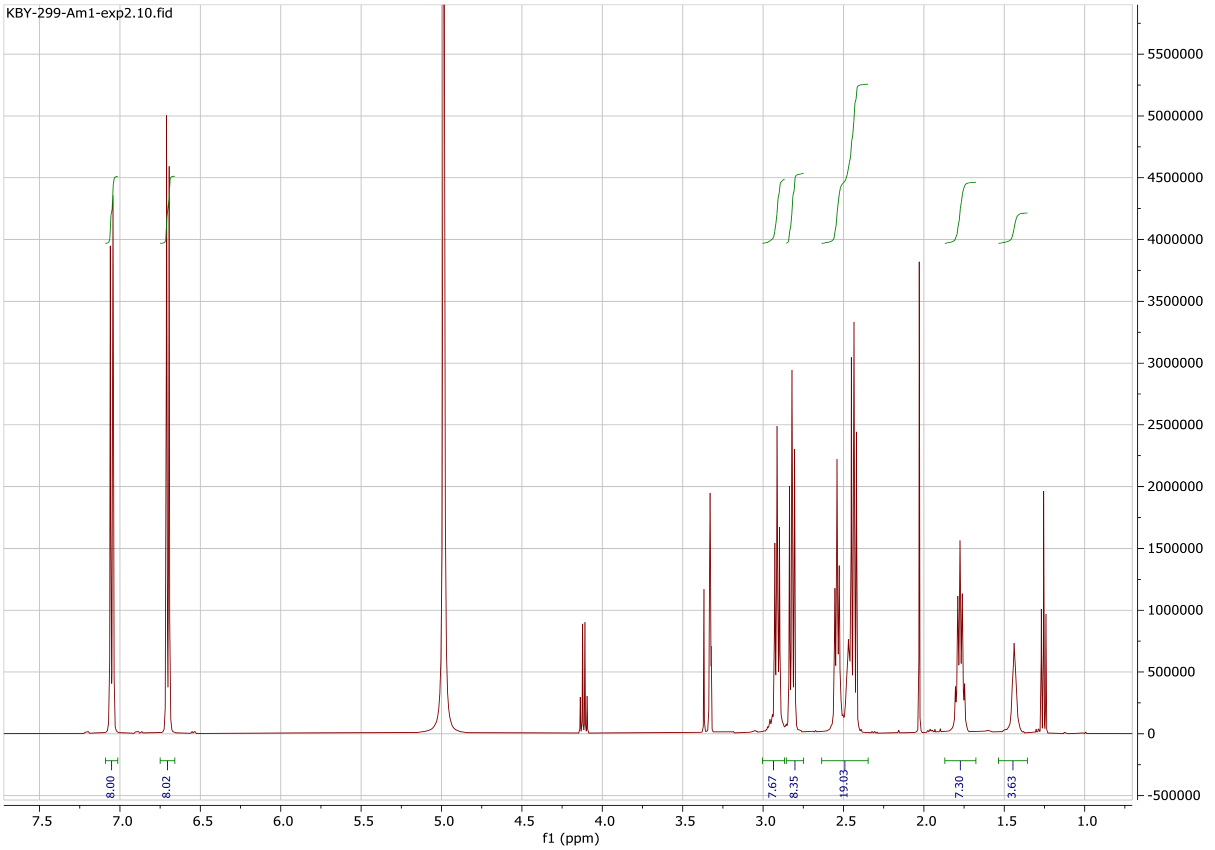

Figure S2 : ^{13}C NMR of PPI-1-PhA in CD_3OD

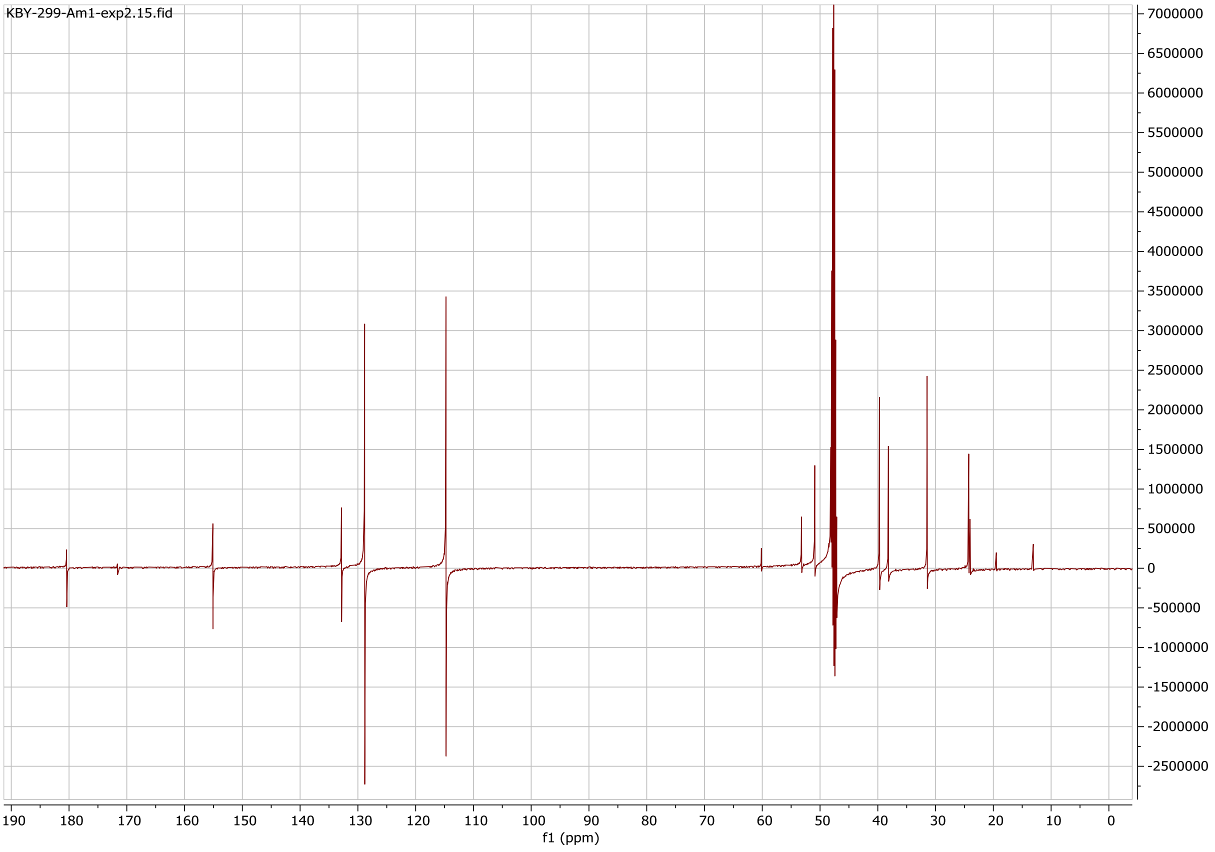

Figure S3 : COSY NMR of PPI-1-PhA in CD_3OD

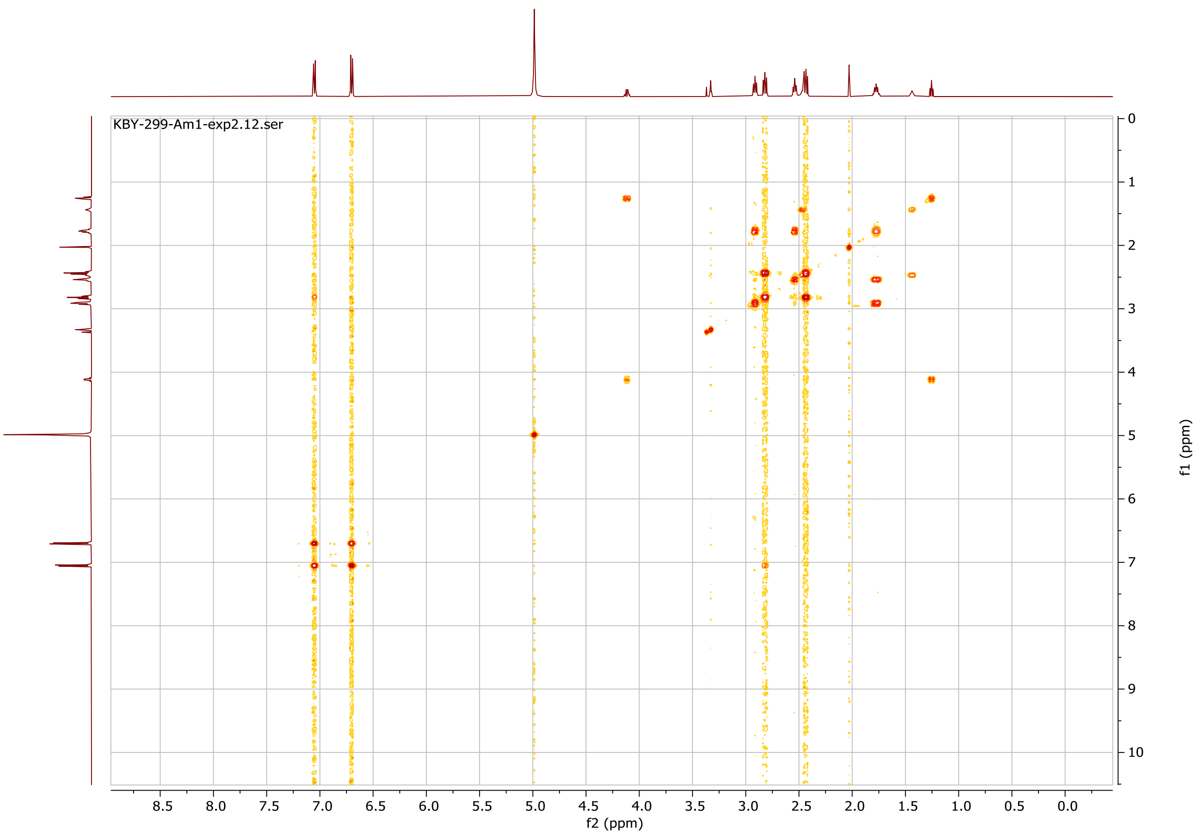

Figure S4 : HSQC NMR of PPI-1-PhA in CD_3OD

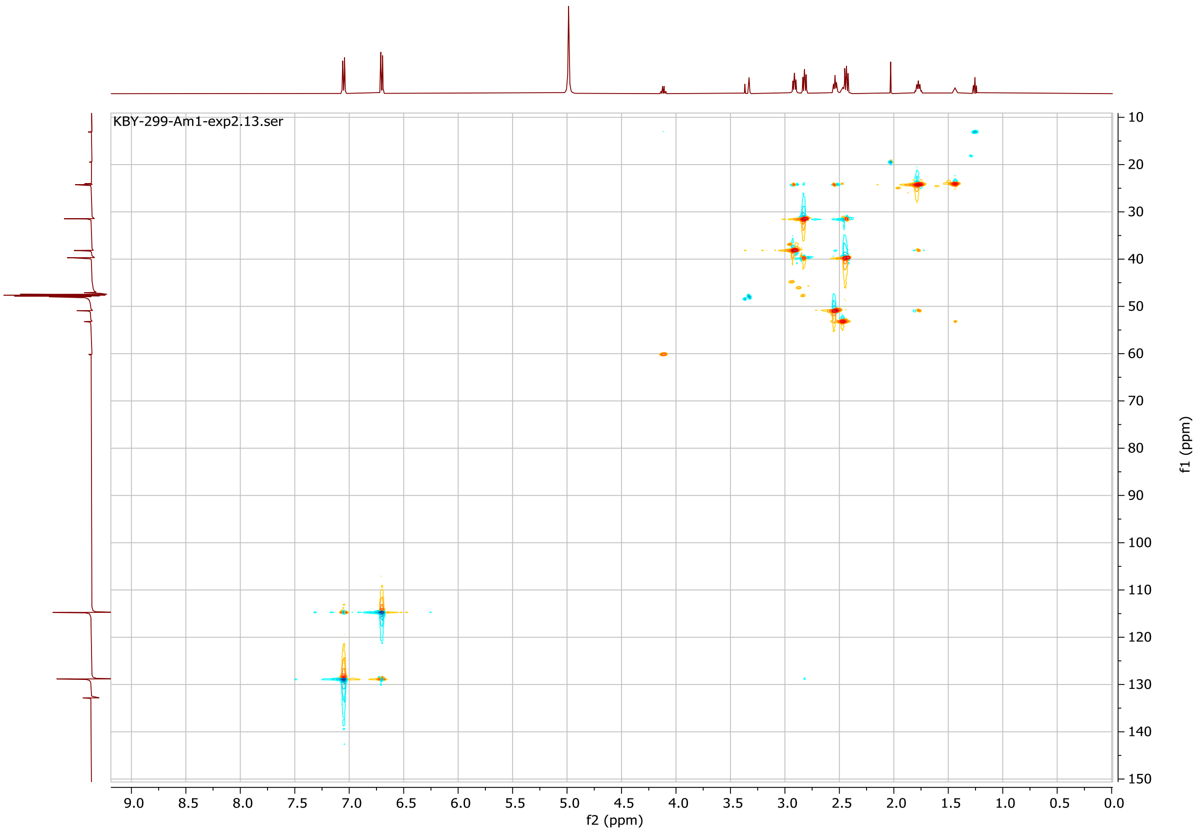

Figure S5: HMBC NMR of PPI-1-PhA in CD_3OD

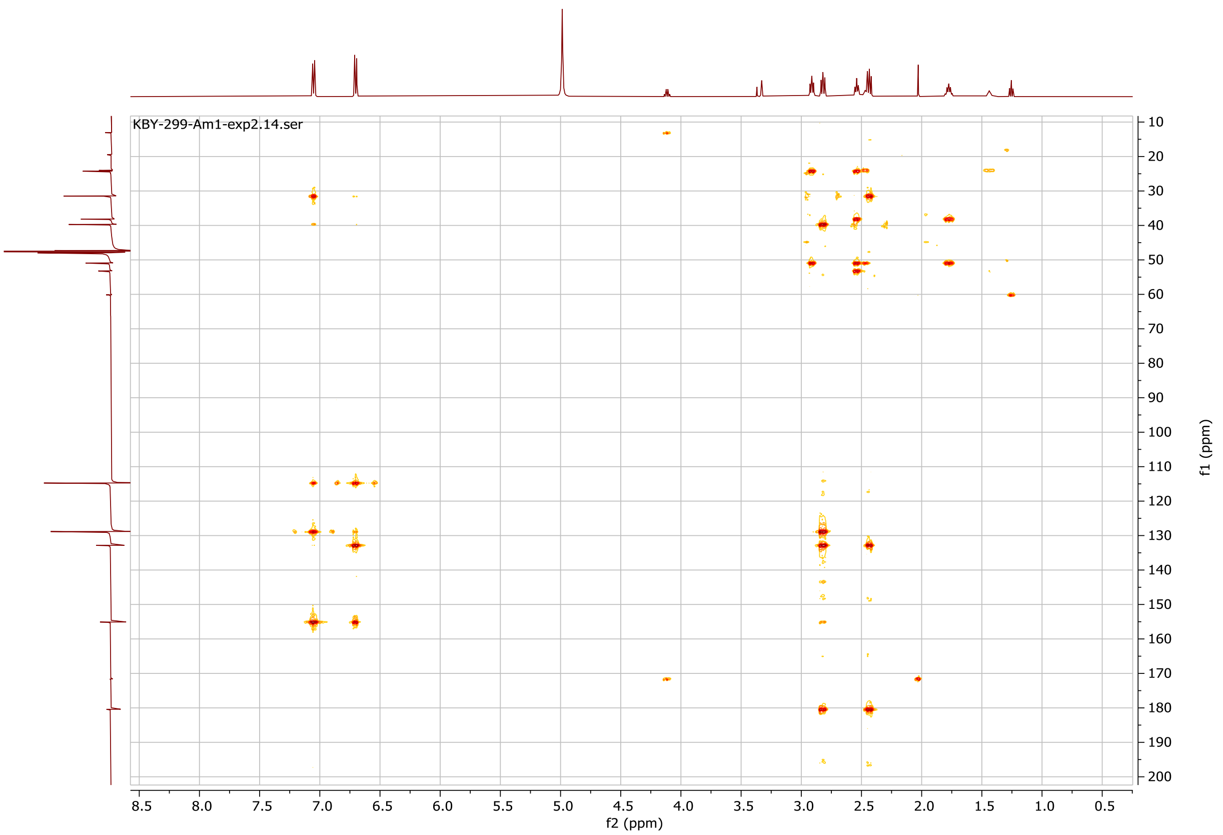

Figure S6: ^{1}H NMR of PPI-2-PhA in CD_3OD

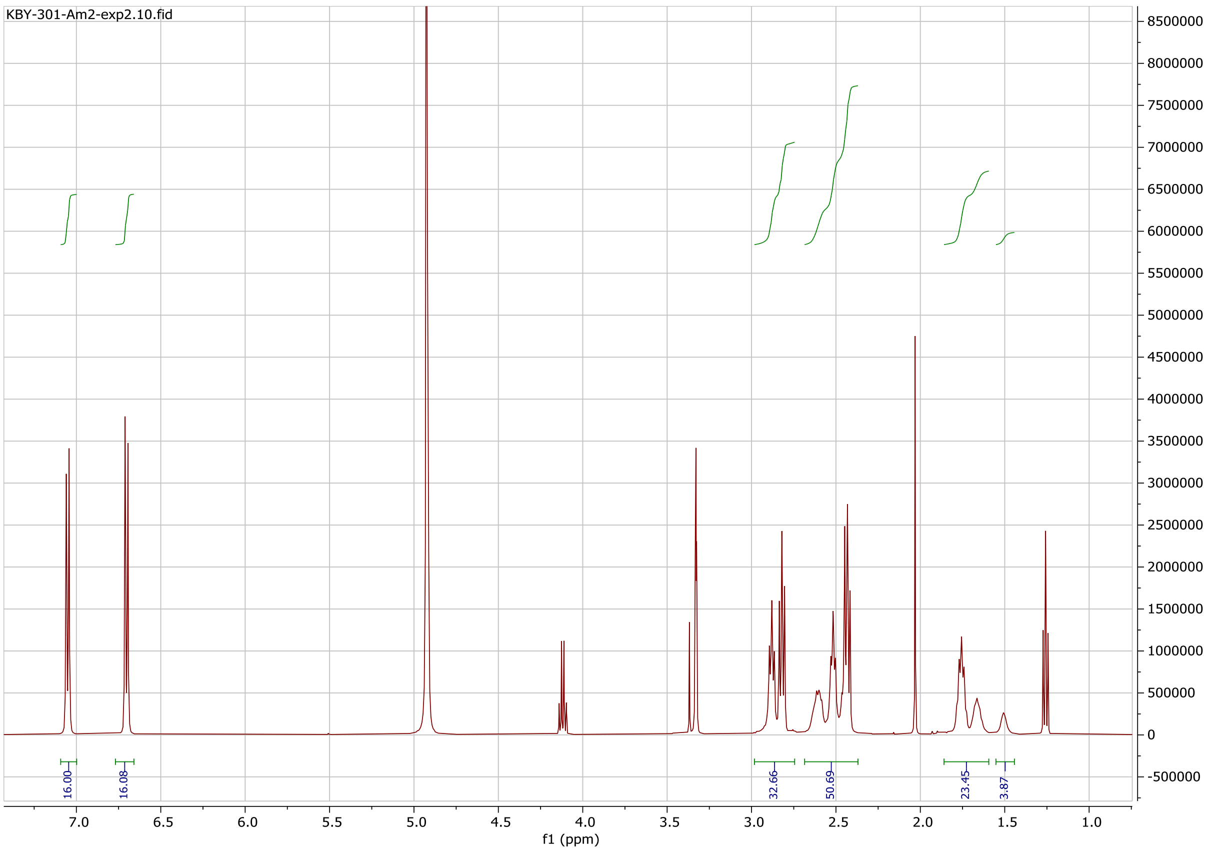

Figure S7: ^{13}C NMR of PPI-2-PhA in CD_3OD

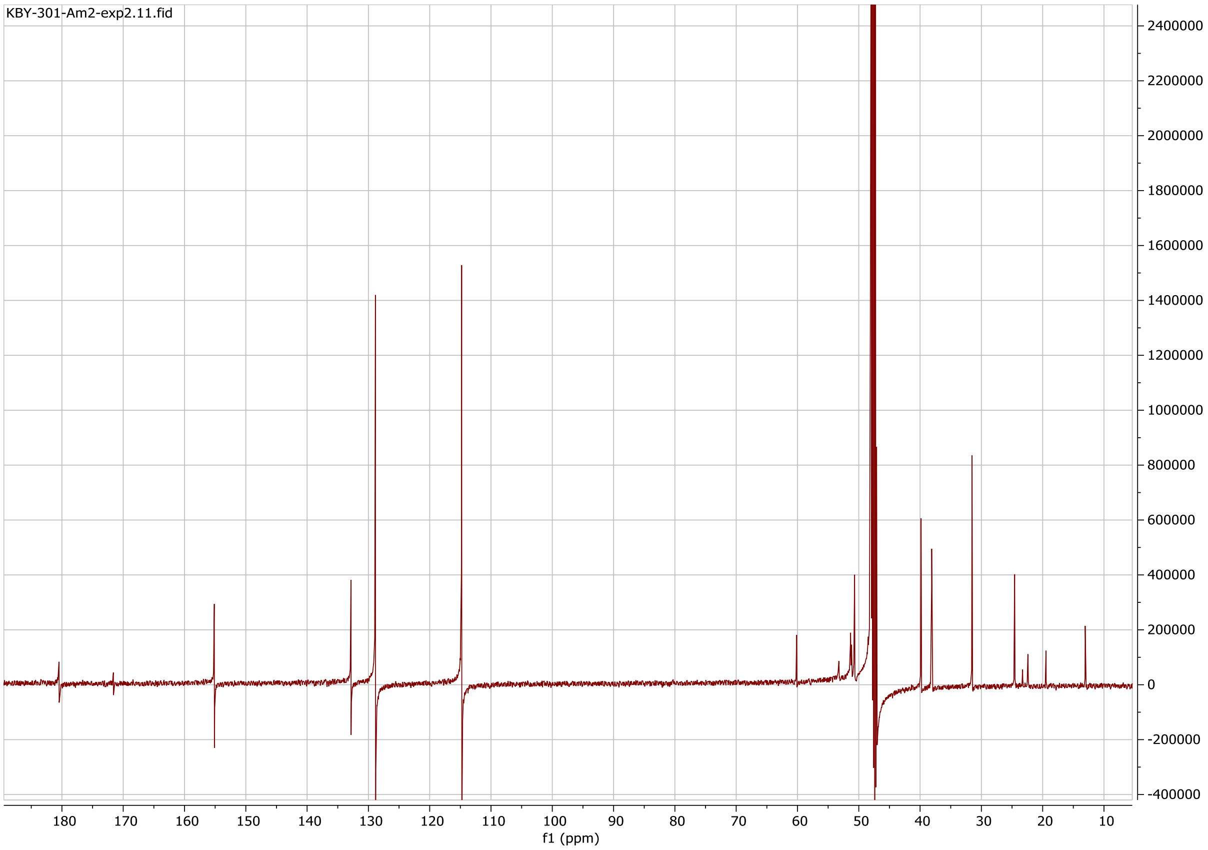

Figure S8: ^{1}H NMR of PPI-3-PhA in CD_3OD

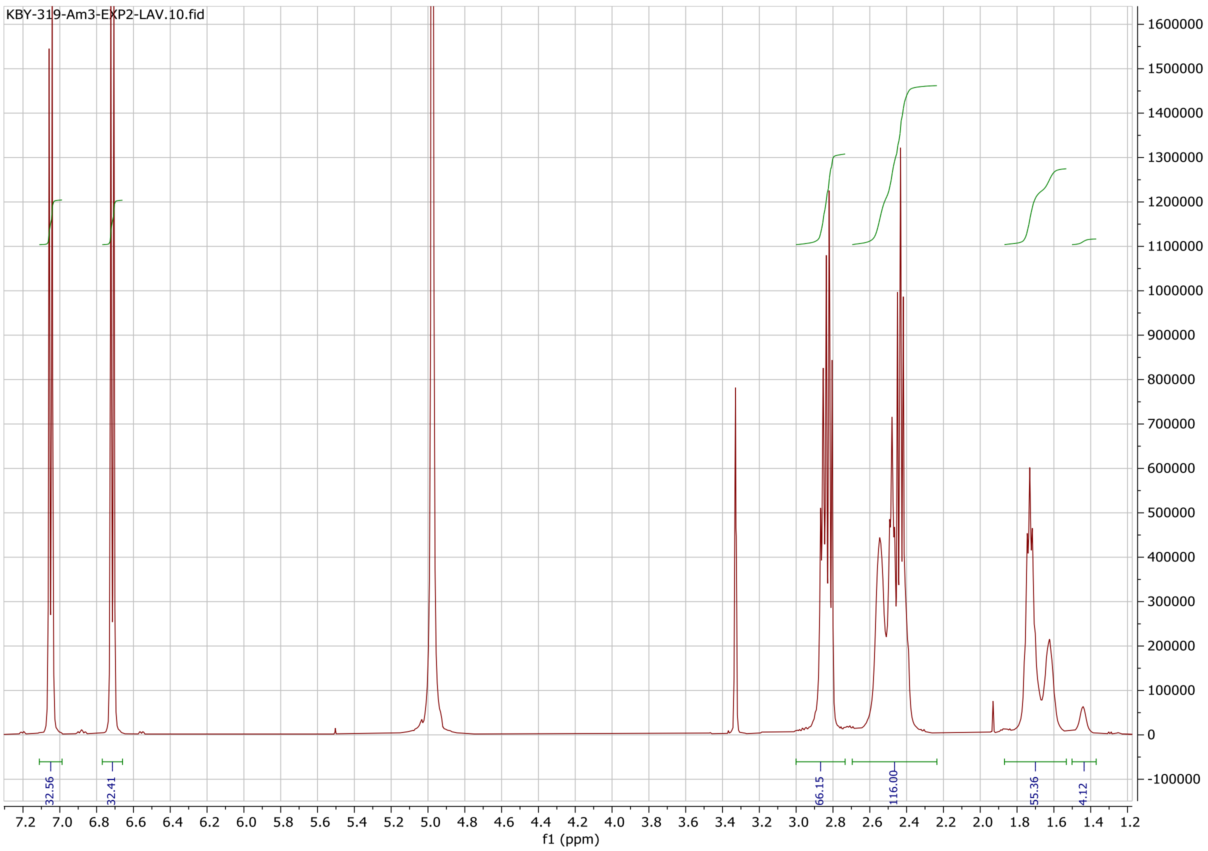

Figure S9: ^{1}H NMR of PPI-1-FA in CD_3OD

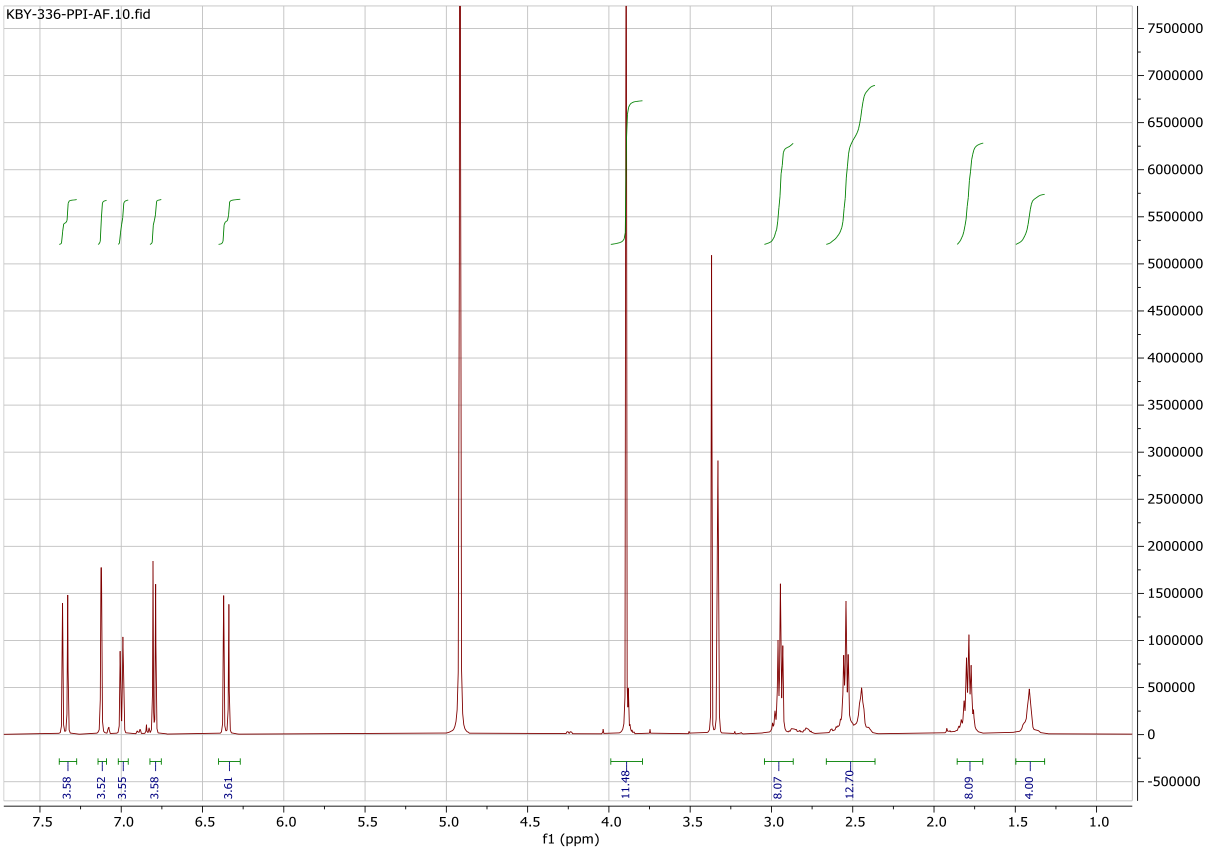

Figure S10: ^{13}C NMR of PPI-1-FA in CD_3OD

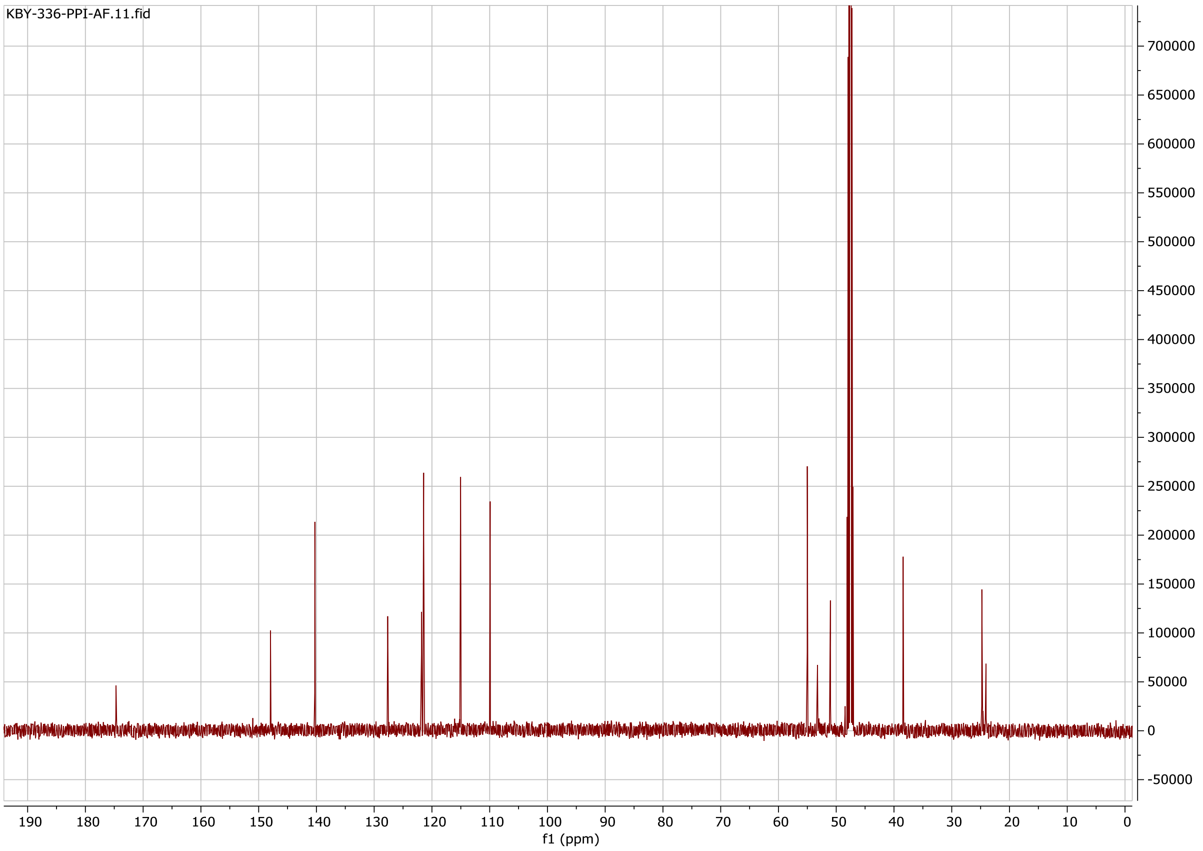

Figure S11: COSY NMR of PPI-1-FA in CD_3OD

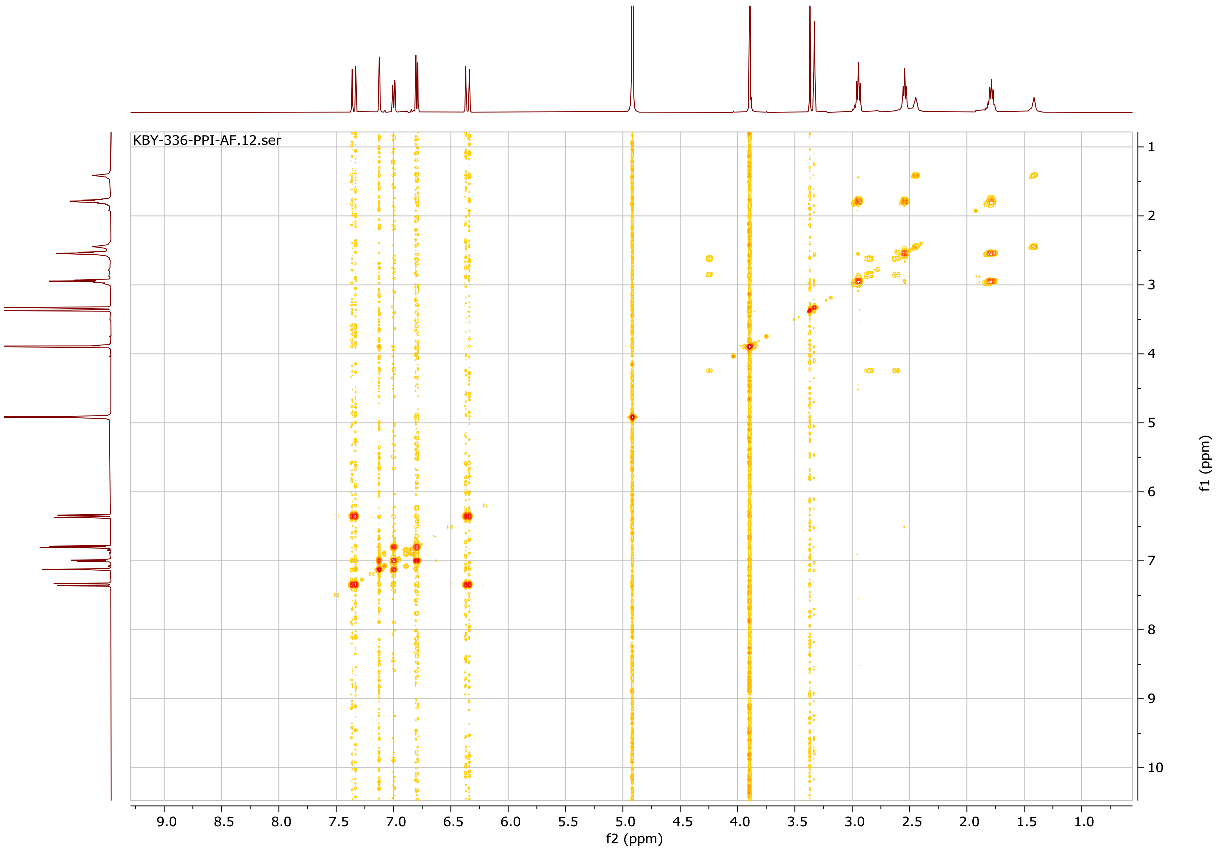

Figure S12: HSQC NMR of PPI-1-FA in CD_3OD

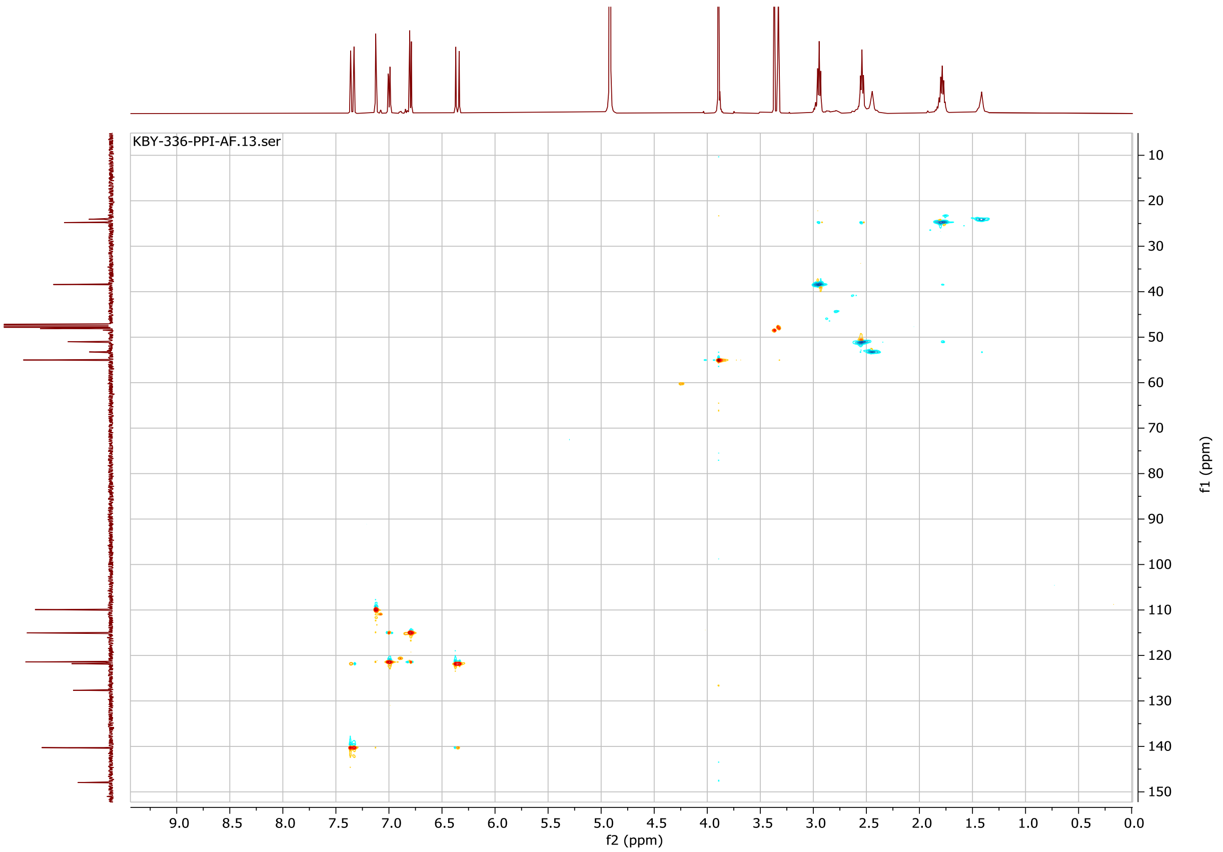

Figure S13: HMBC NMR of PPI-1-FA in CD_3OD

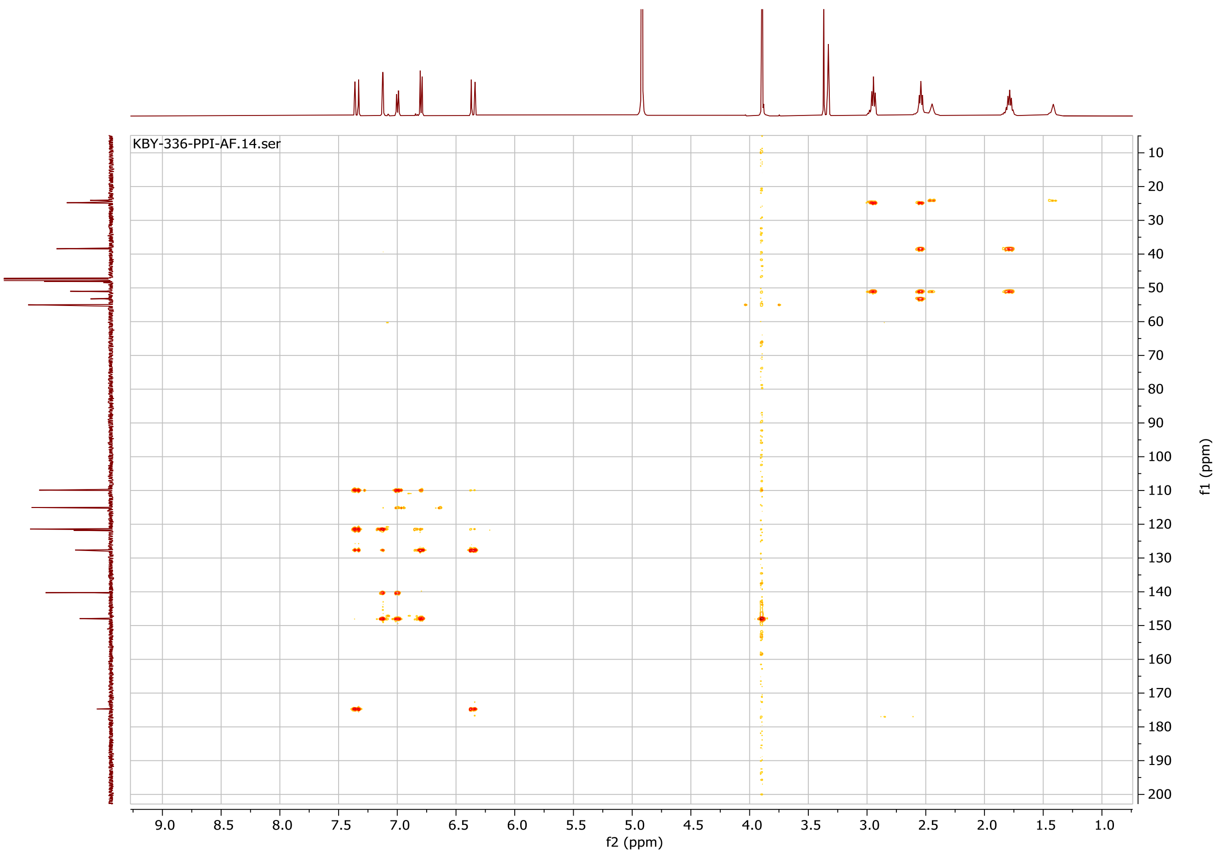

Figure S14: ^1H NMR of PPI-2-FA in CD_3OD

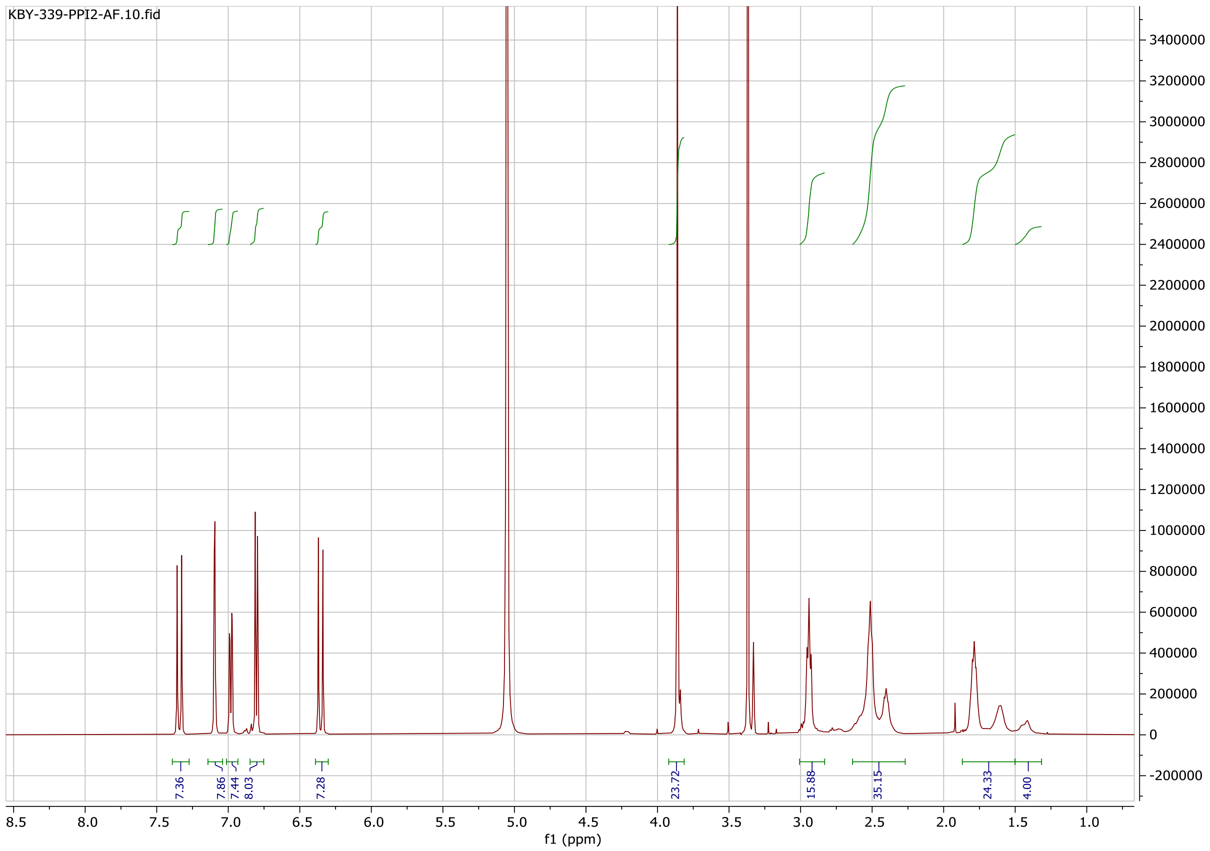

Figure S15: ^{13}C NMR of PPI-2-FA in CD_3OD

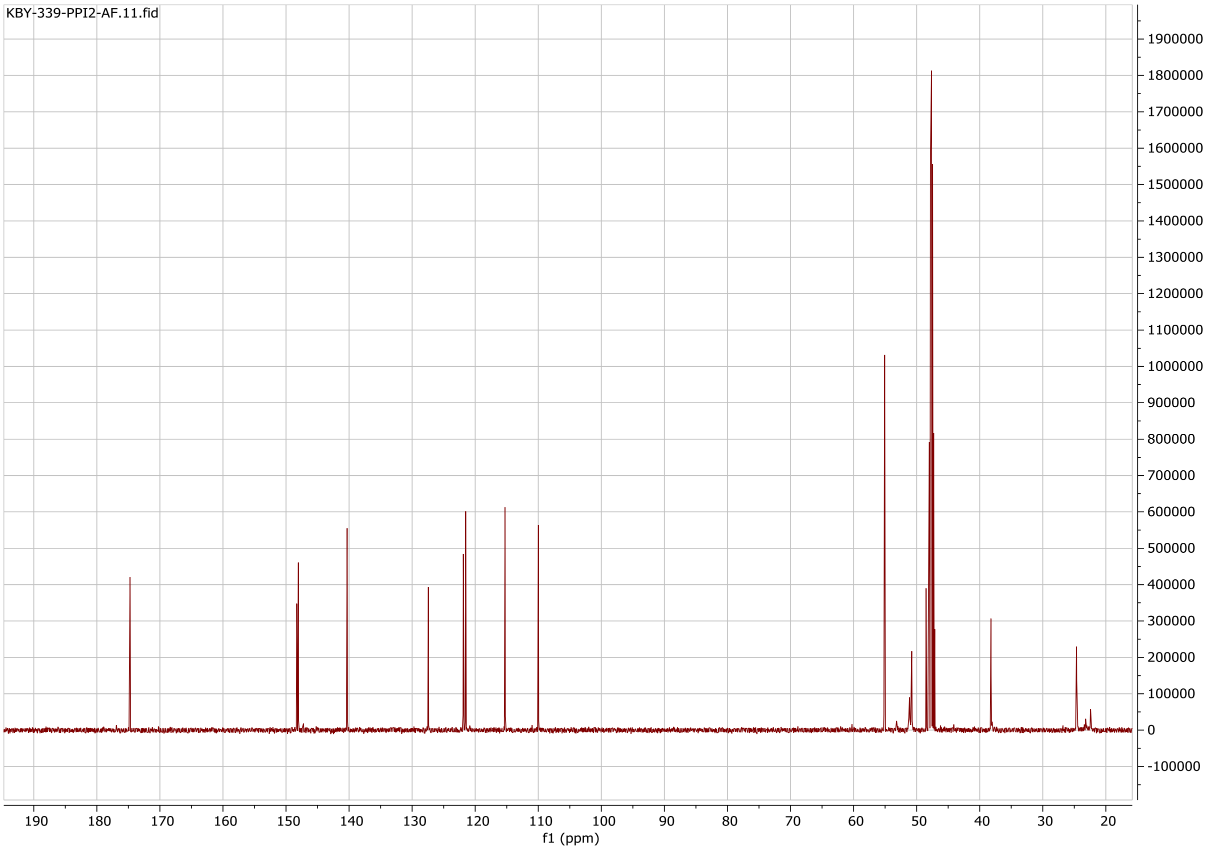

Figure S16: ^{1}H NMR of PPI-3-FA in CD_3OD

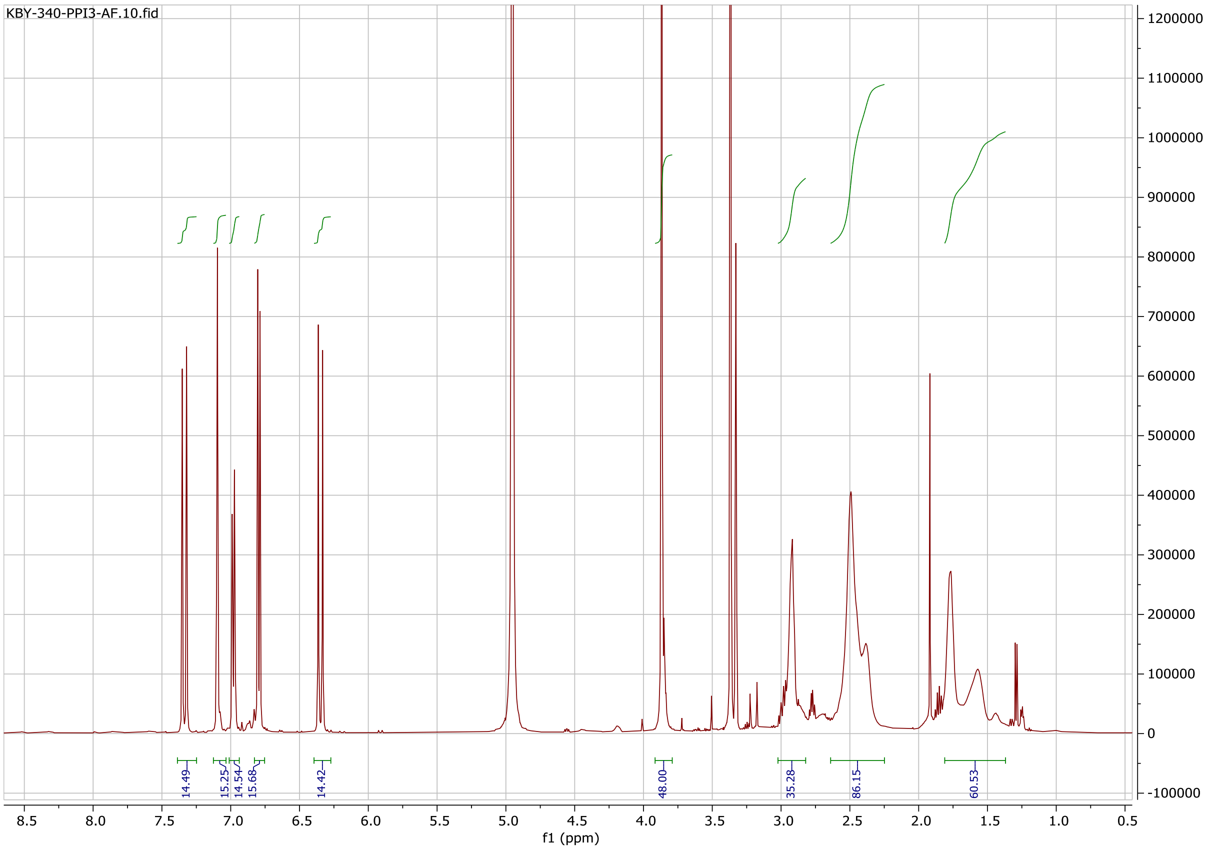

Figure S17: ^{13}C NMR of PPI-3-FA in CD_3OD

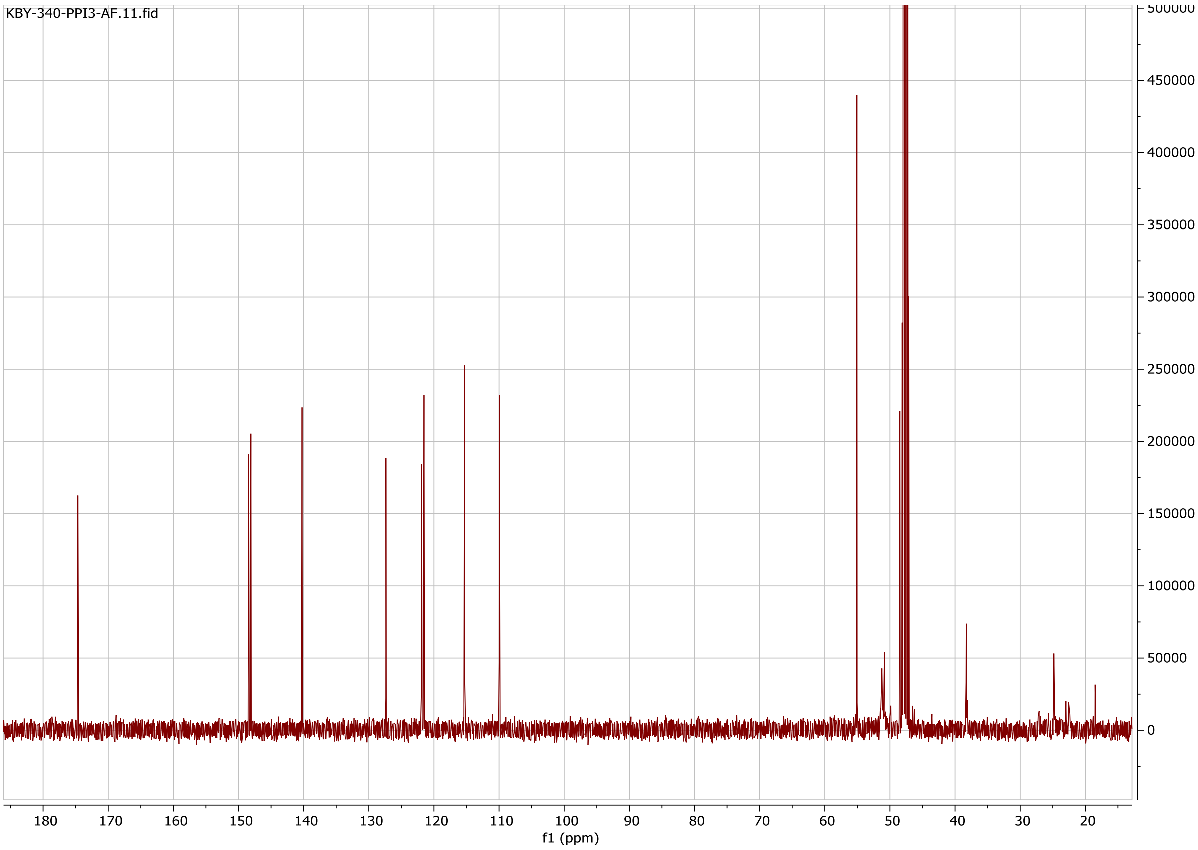

Figure S18: IR of PPI-3-FA

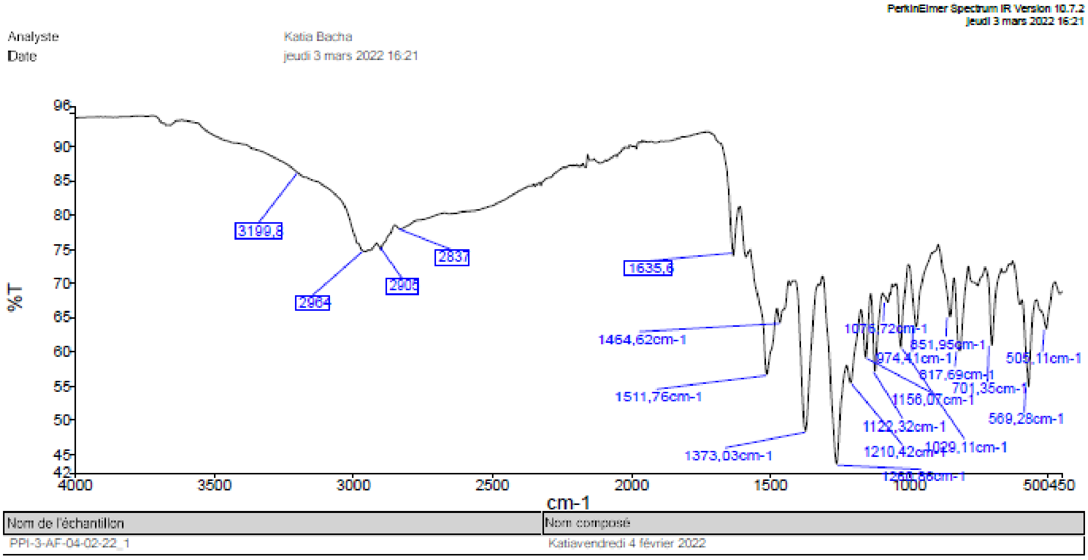

Figure S19: ^{1}H NMR of PPI-1-CA in $(CD_3)_2SO$

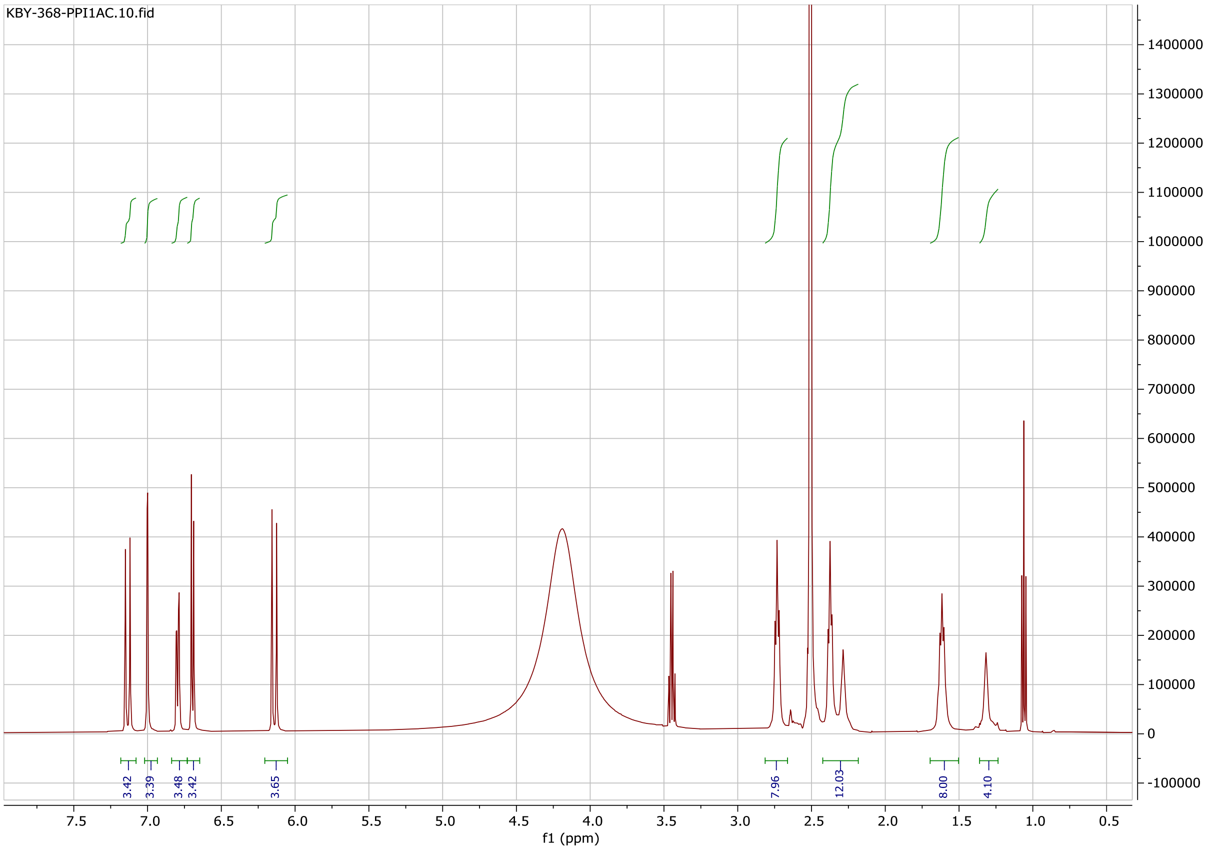

Figure S20: ^{13}C NMR of PPI-1-CA in $(CD_3)_2SO$

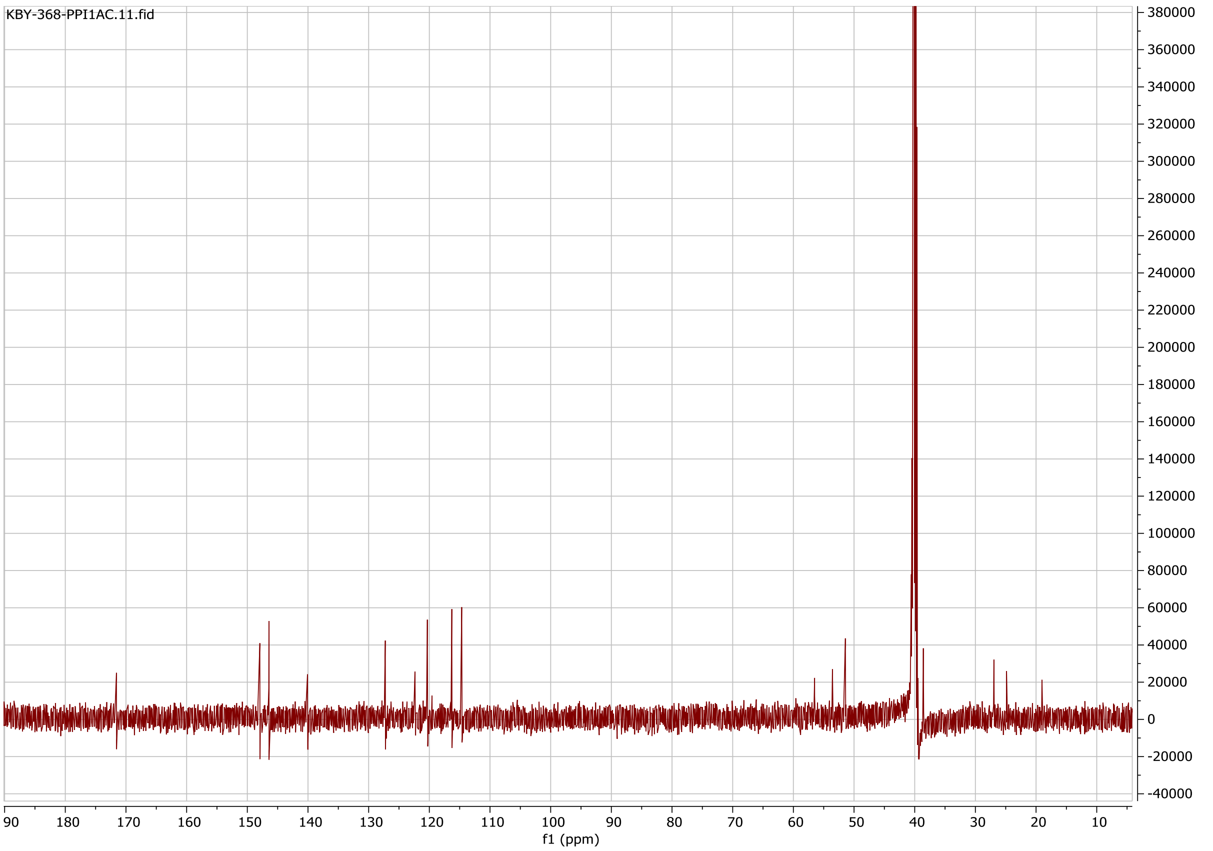

Figure S21: ^{1}H NMR of PPI-2-CA in $(CD_3)_2SO$

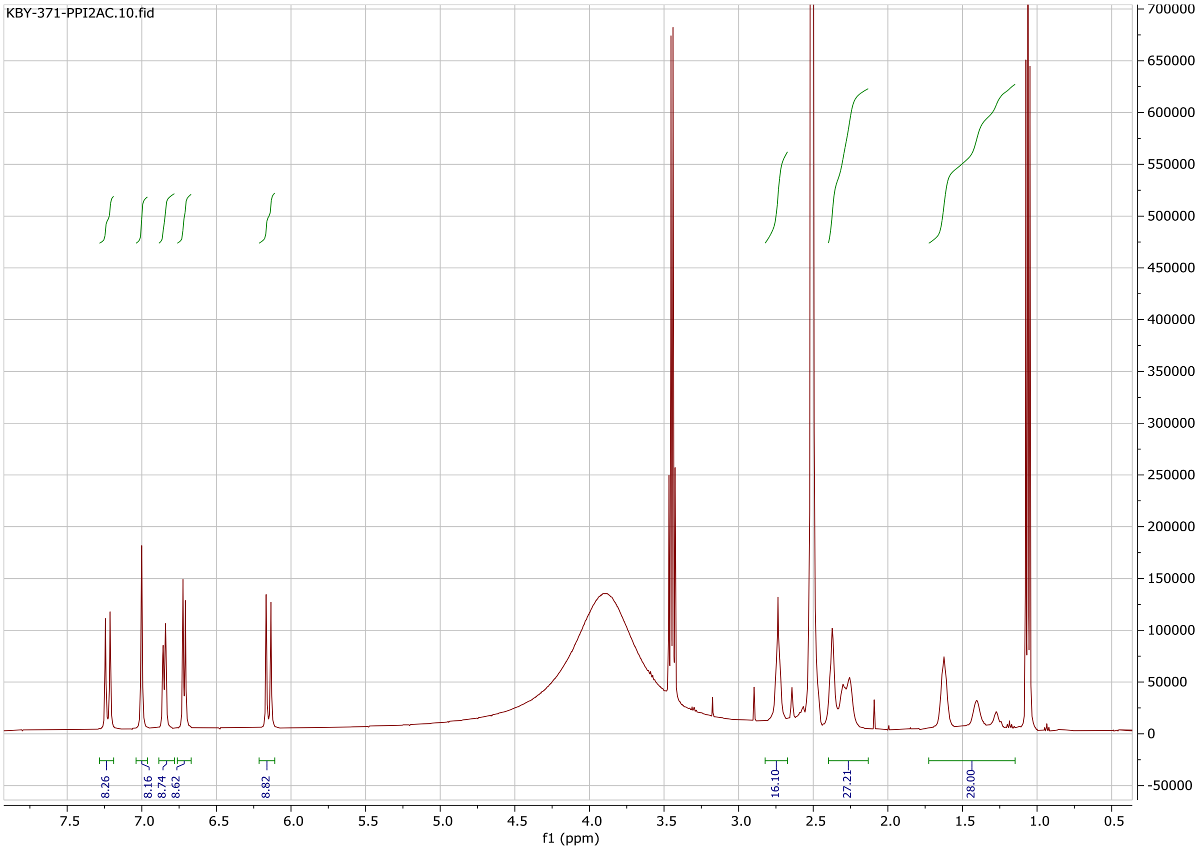

Figure S22: ^{13}C NMR of PPI-2-CA in $(CD_3)_2SO$

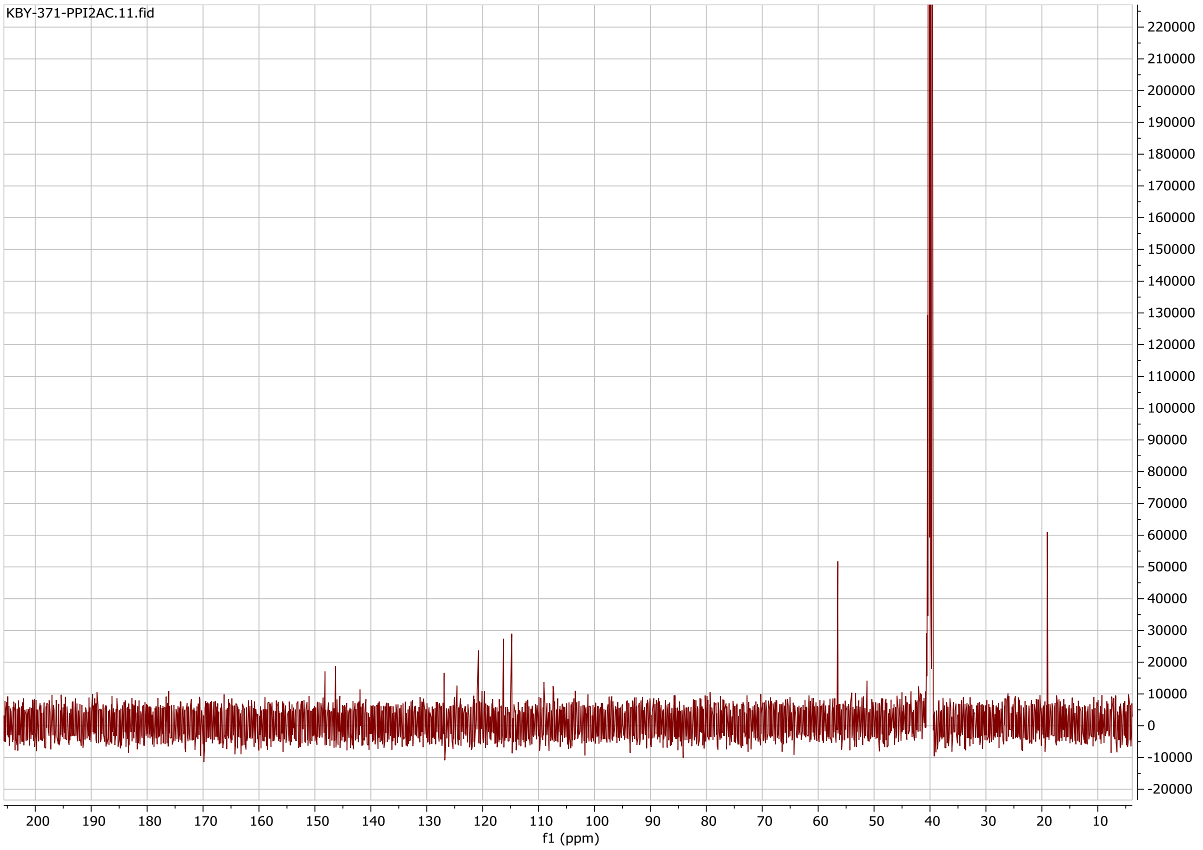

Figure S23: ^{1}H NMR of PPI-3-CA in $(CD_3)_2SO$ (weak solubility)

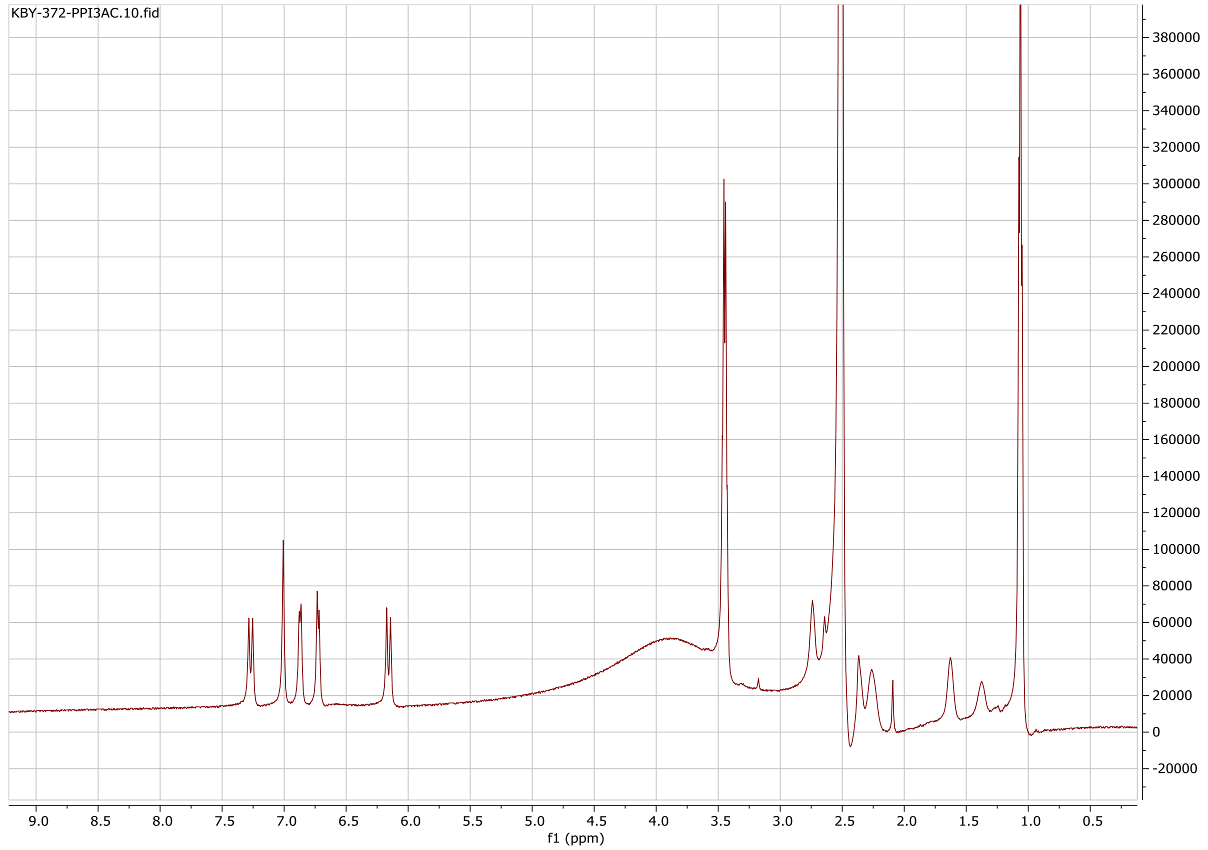

Figure S24: ^{13}C NMR de PPI-3-CA in $(CD_3)_2SO$ (weak solubility)

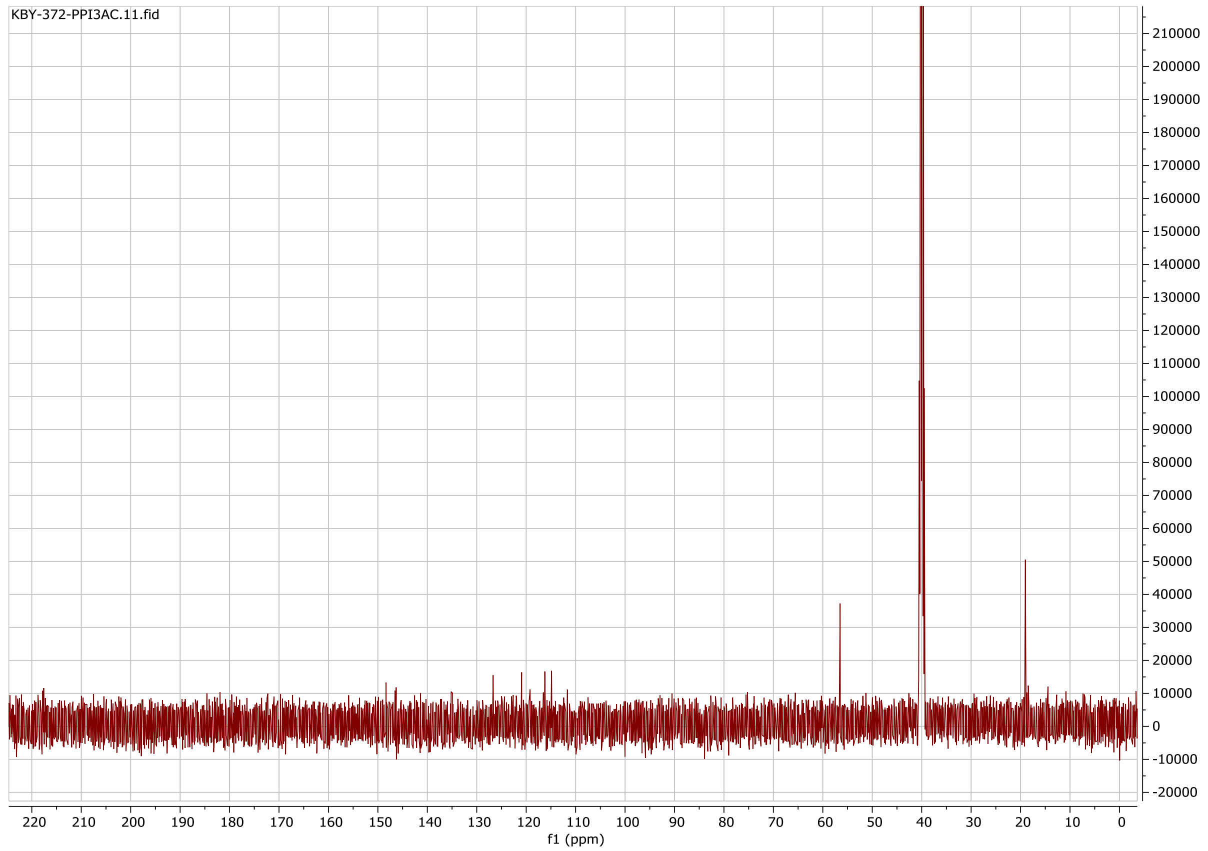

Figure S25: IR of PPI-3-CA

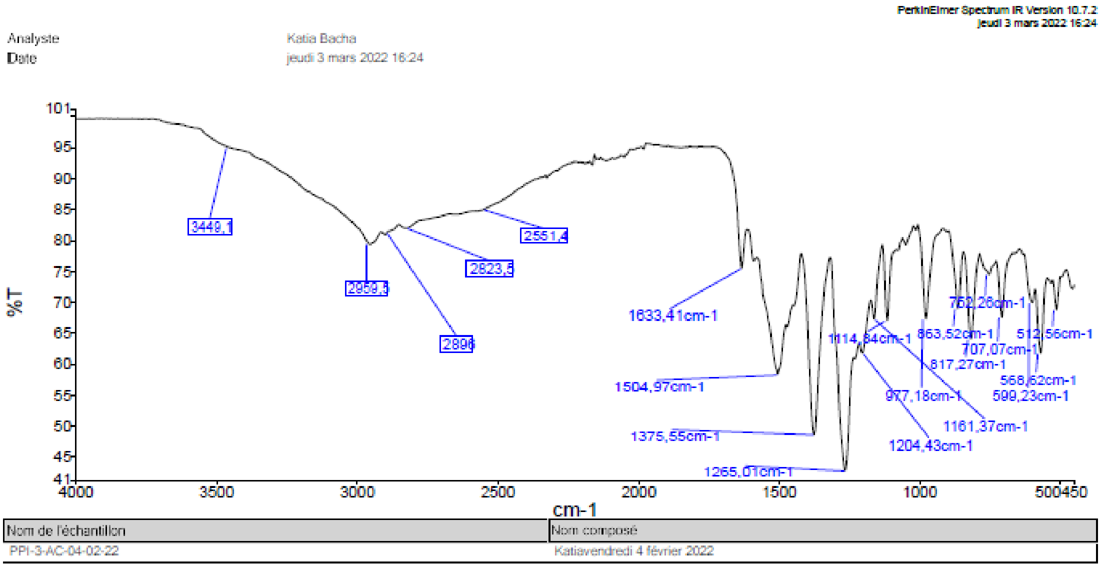

Figure S26: IR of Caffeic acid

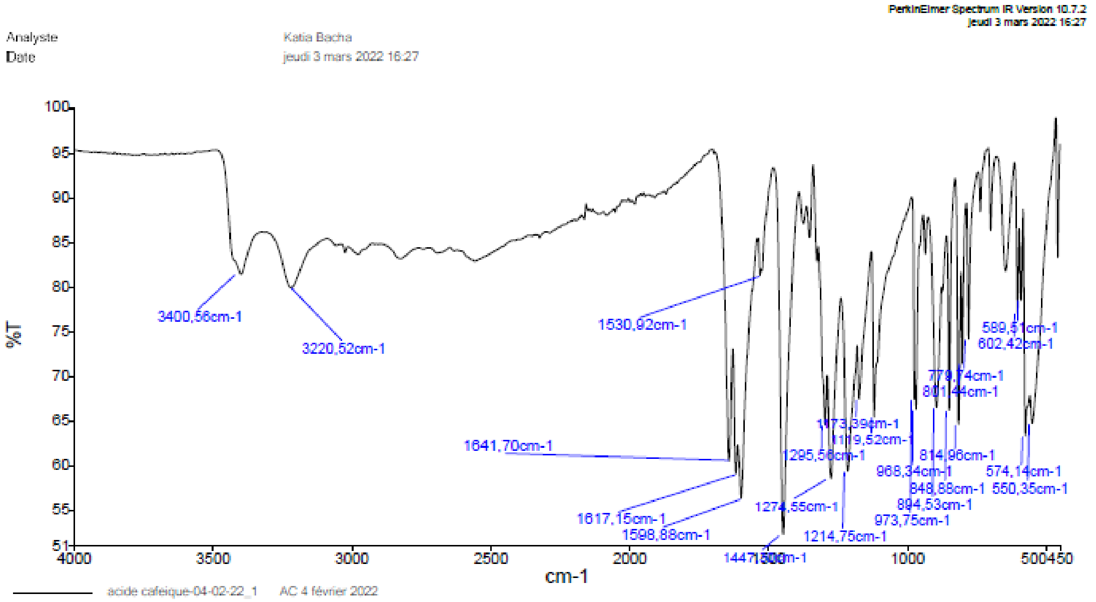

Figure S27: ^{1}H NMR of PAMAM-1-PhA in CD_3OD

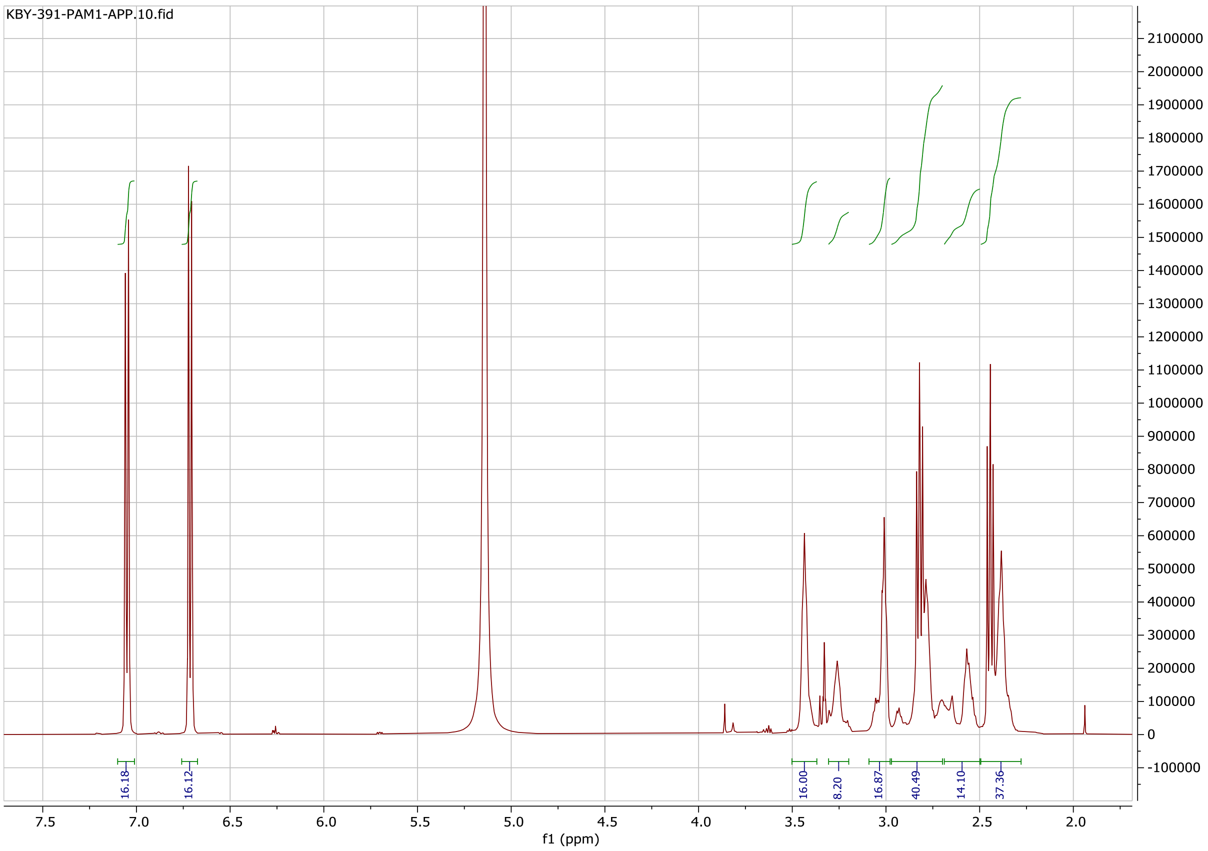

Figure S28: ^{13}C NMR of PAMAM-1-PhA in CD_3OD

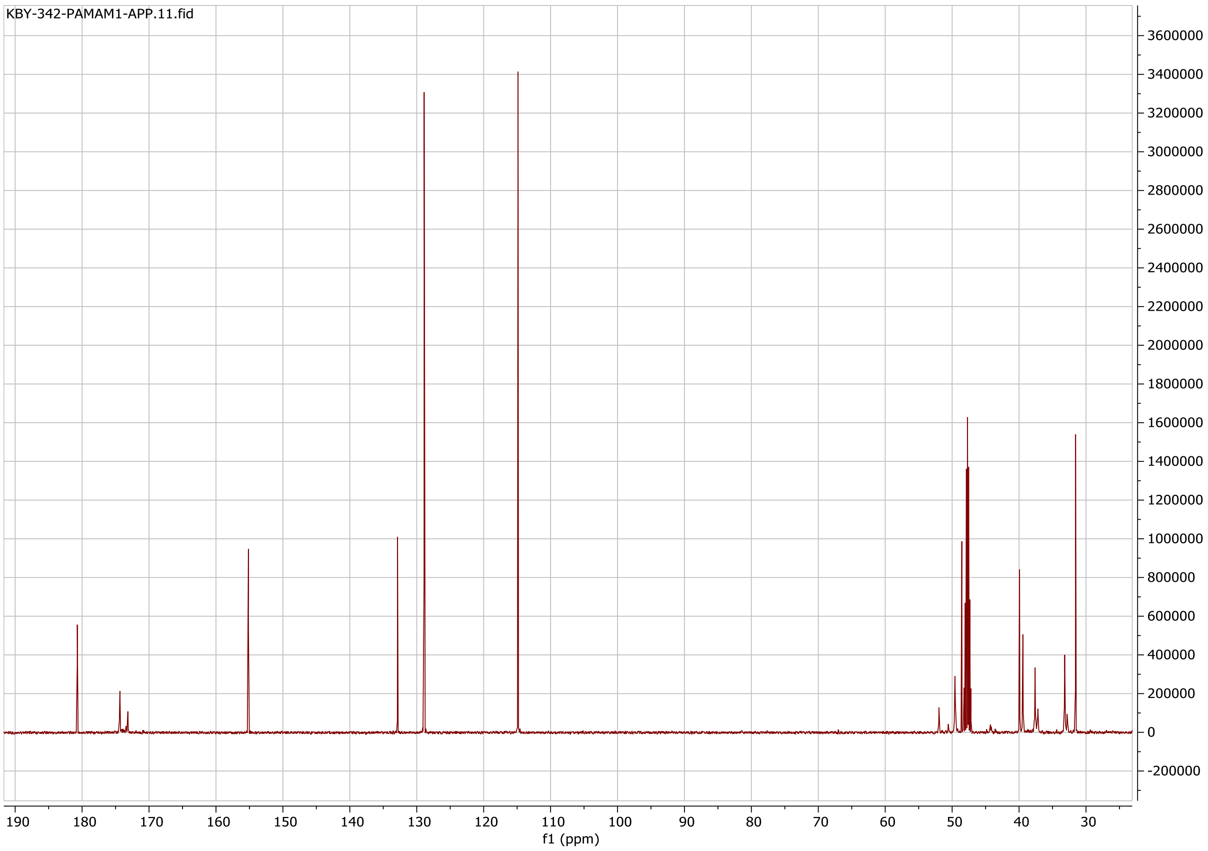

Figure S29: COSY NMR of PAMAM-1-PhA in CD_3OD

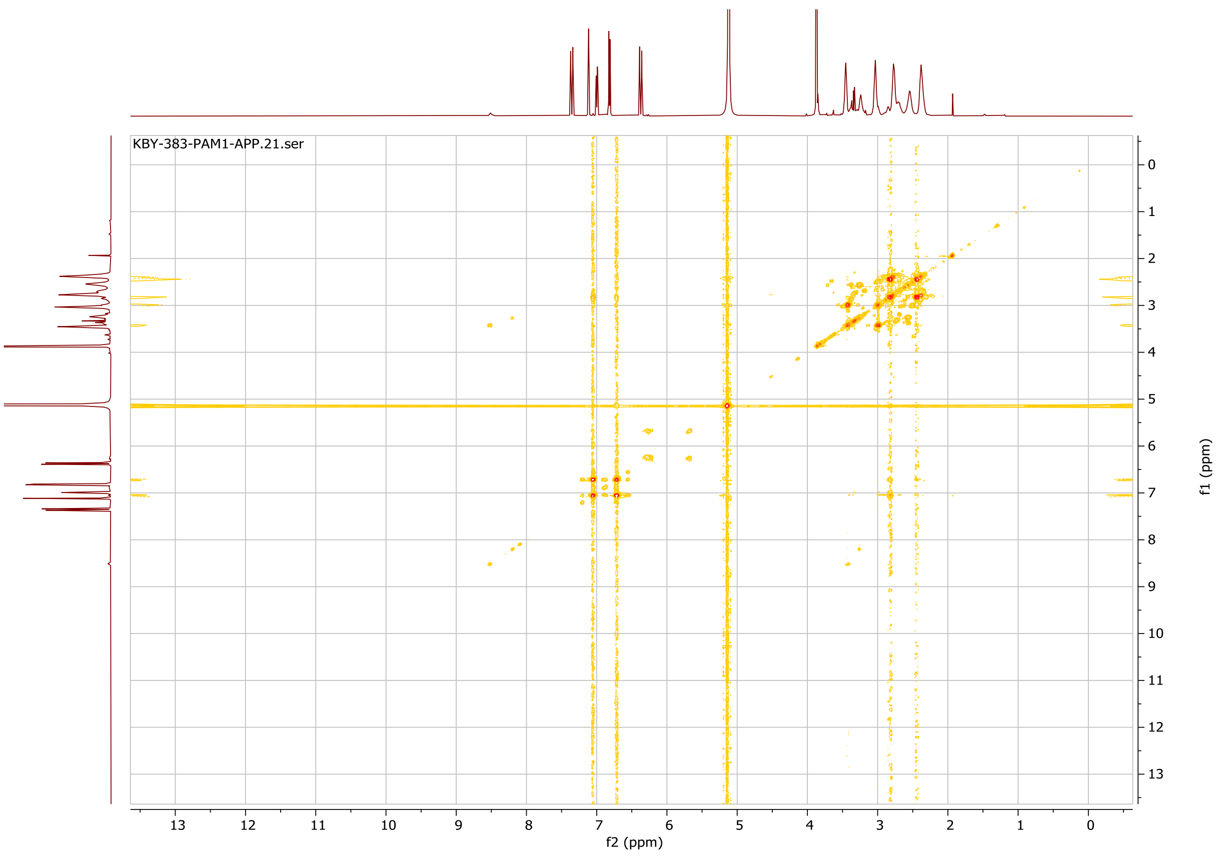

Figure S30: IR of PAMAM-1-PhA

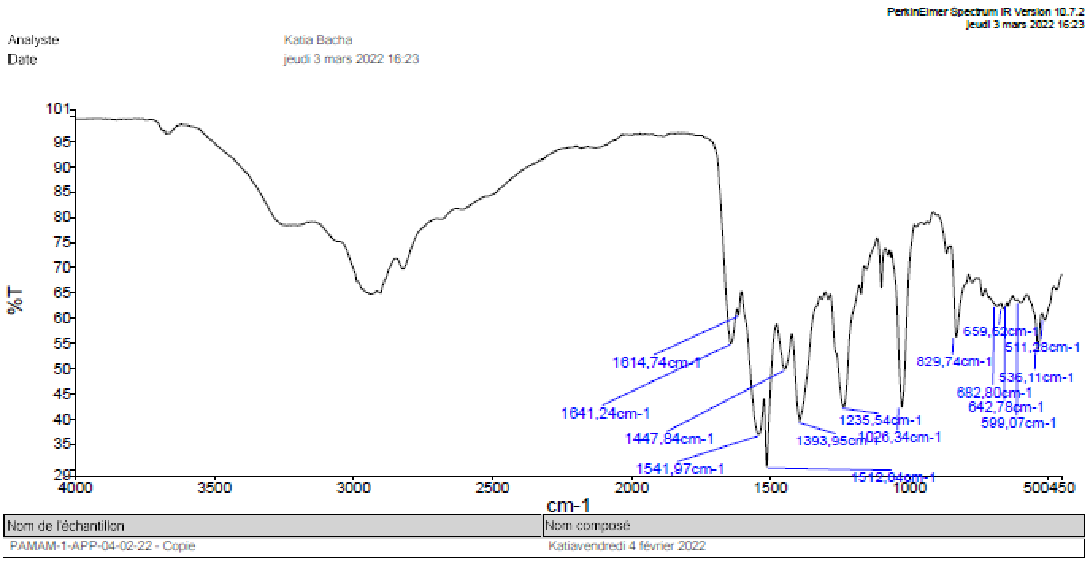

Figure S31: IR of Phloretic acid

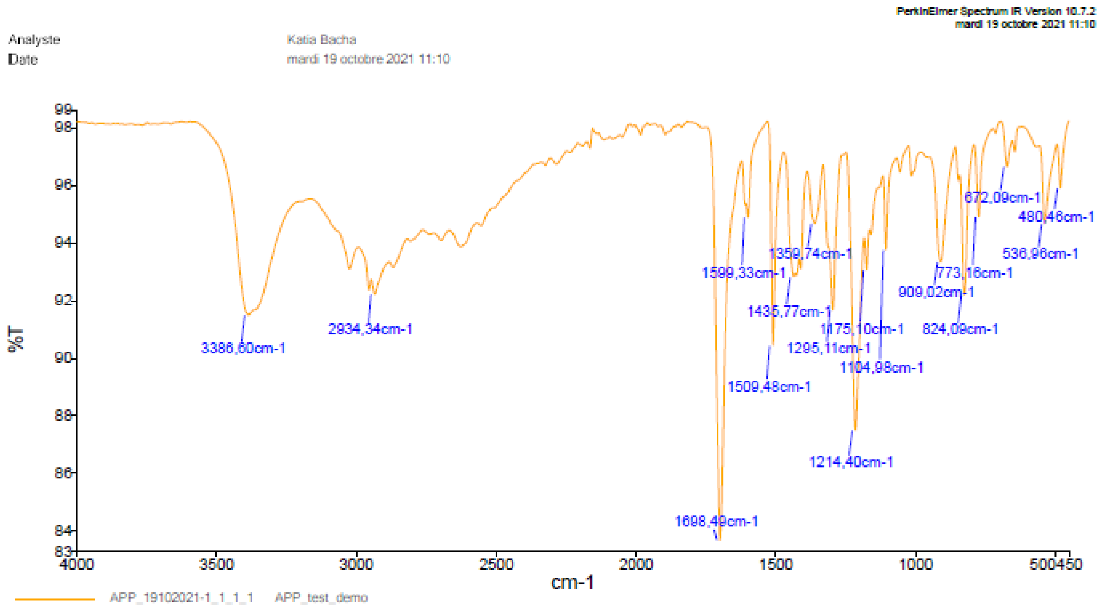

Résultats spectre source	
Nom du spectre	Nombre de Pics
APP_19102021-1_1_1_1	17

Page 1

Figure S32: ^{1}H NMR of PAMAM-2-PhA in CD_3OD

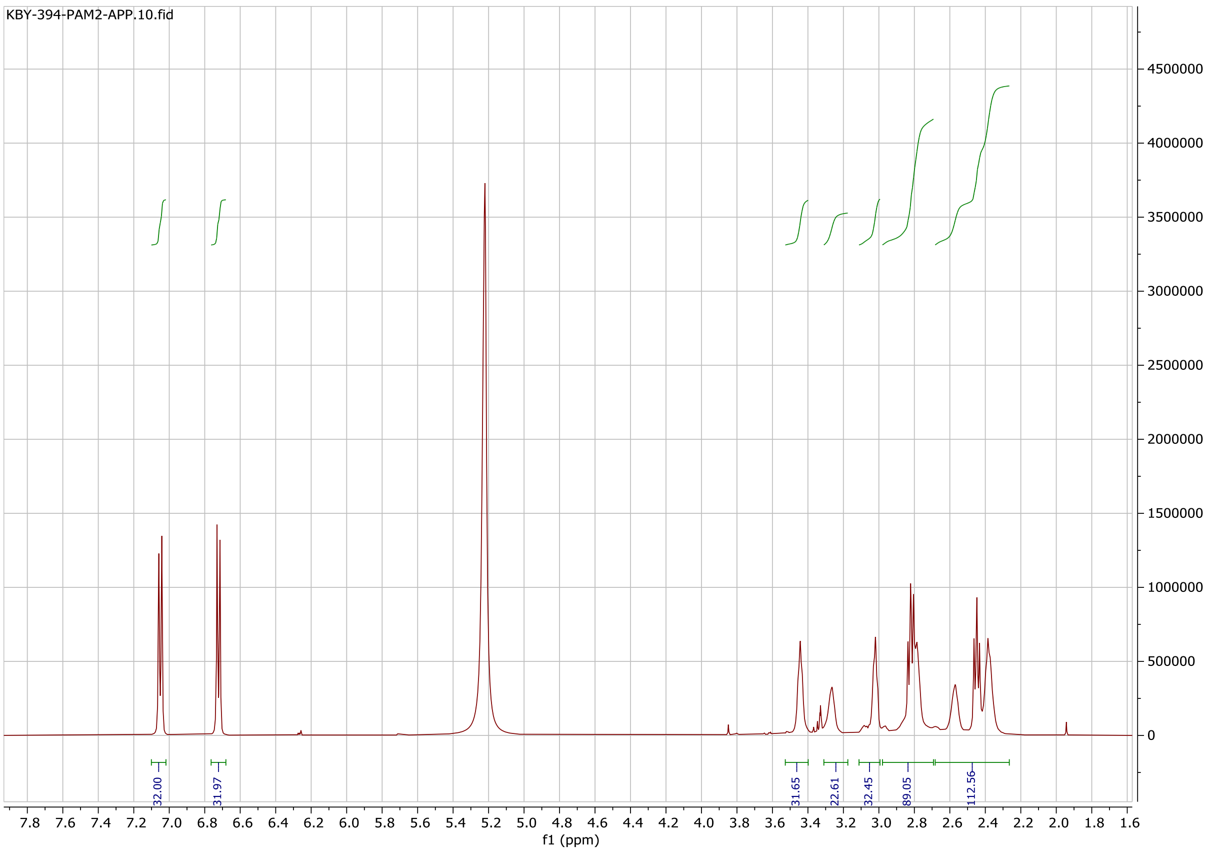

Figure S33: IR of PAMAM-2-PhA

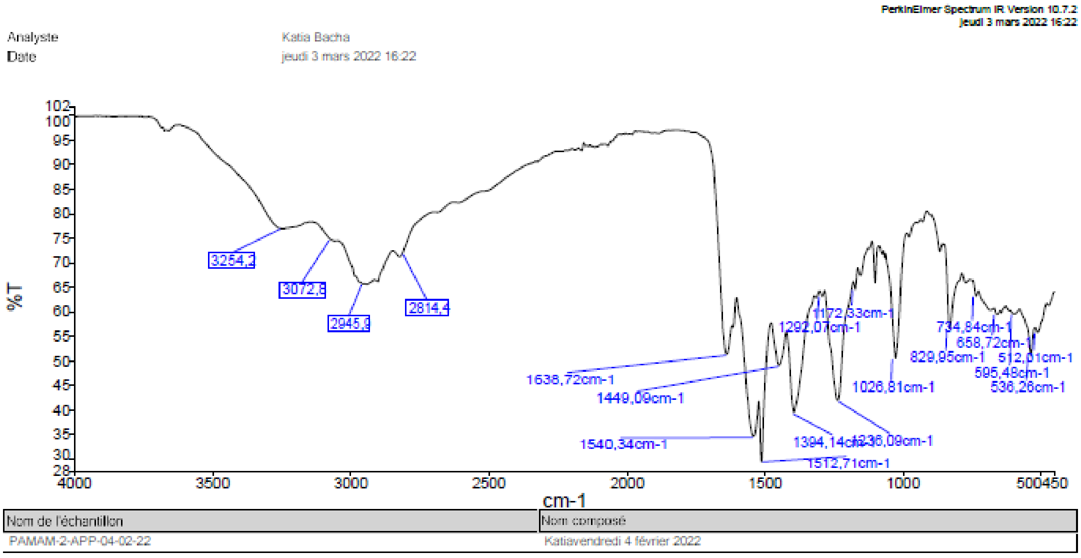

Figure S34: ^{1}H NMR of PAMAM-3-PhA in CD_3OD

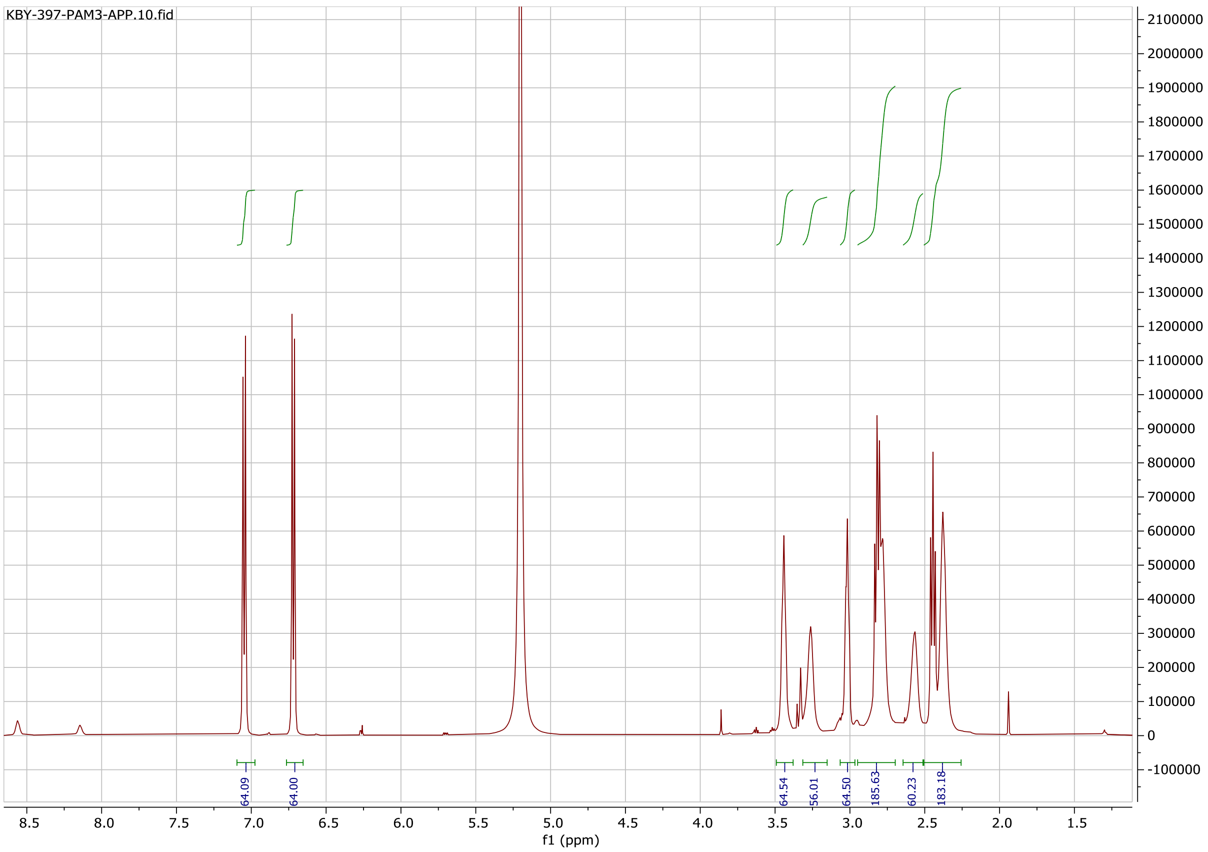

Figure S35: ^{13}C NMR of PAMAM-3-PhA in CD_3OD

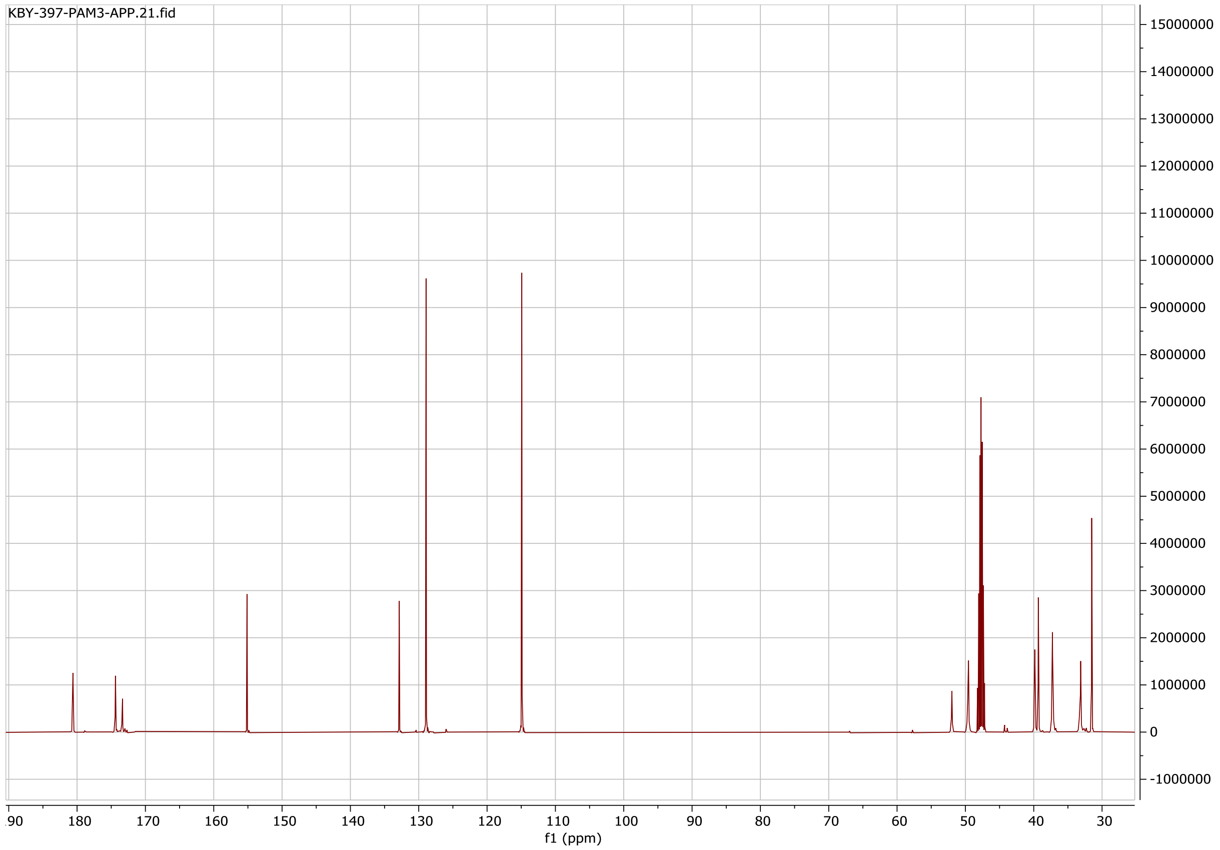

Figure S36: HMBC NMR of PAMAM-3-PhA in CD_3OD

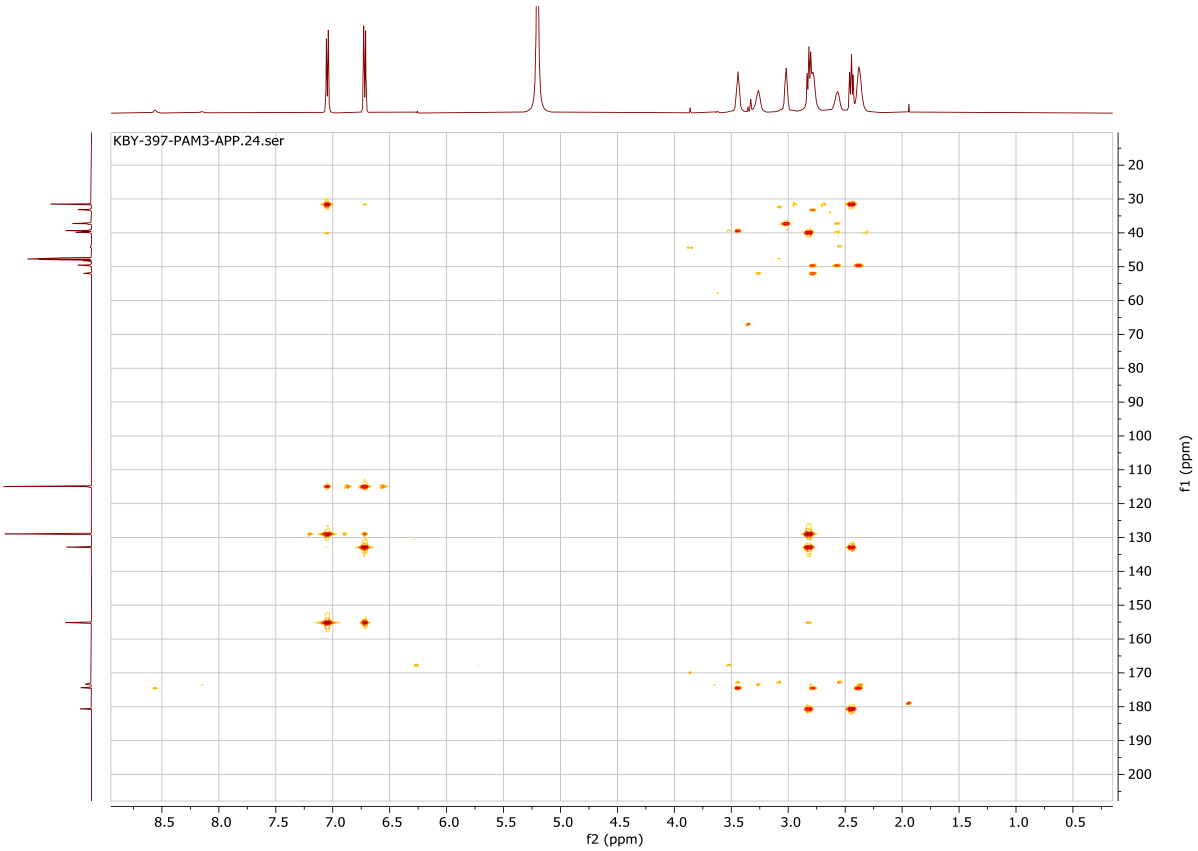

Figure S37: ^{1}H NMR of PAMAM-1-FA in CD_3OD

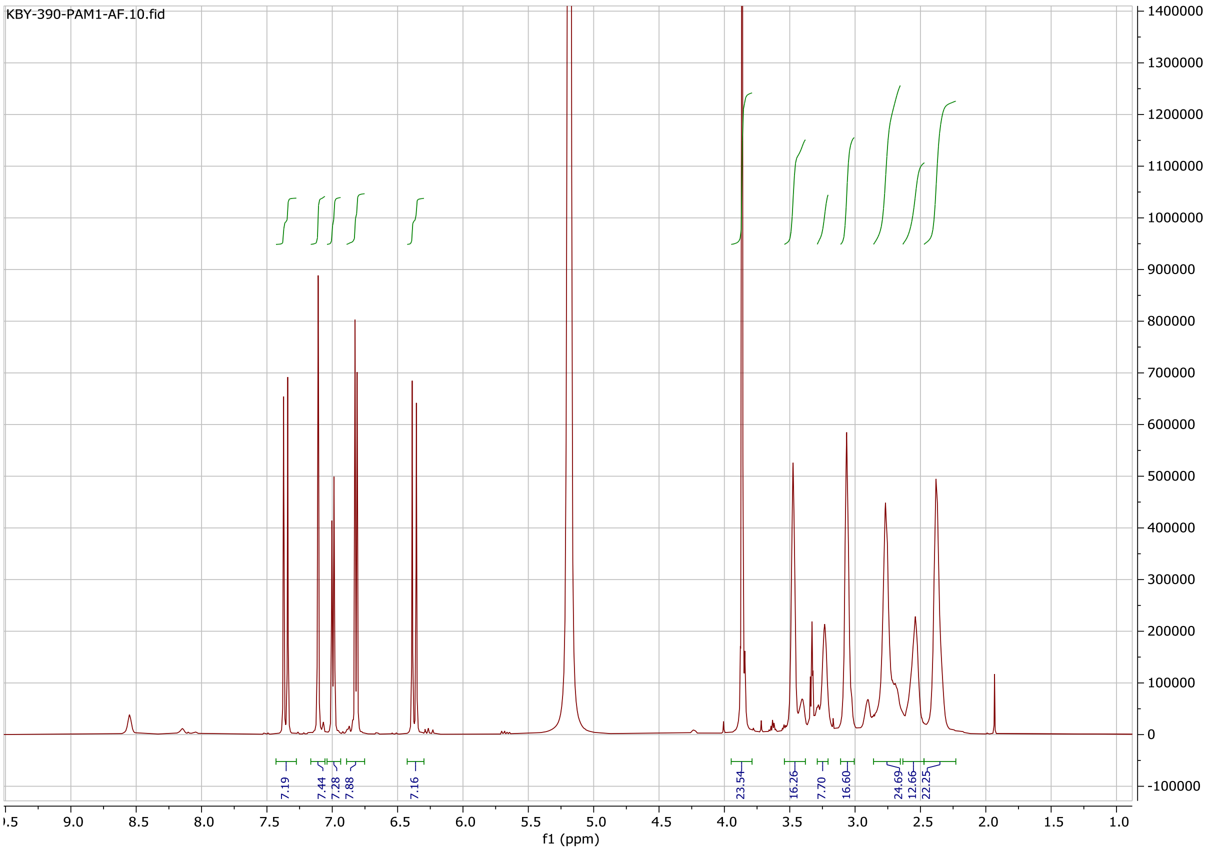

Figure S38: ^{13}C NMR of PAMAM-1-FA in CD_3OD

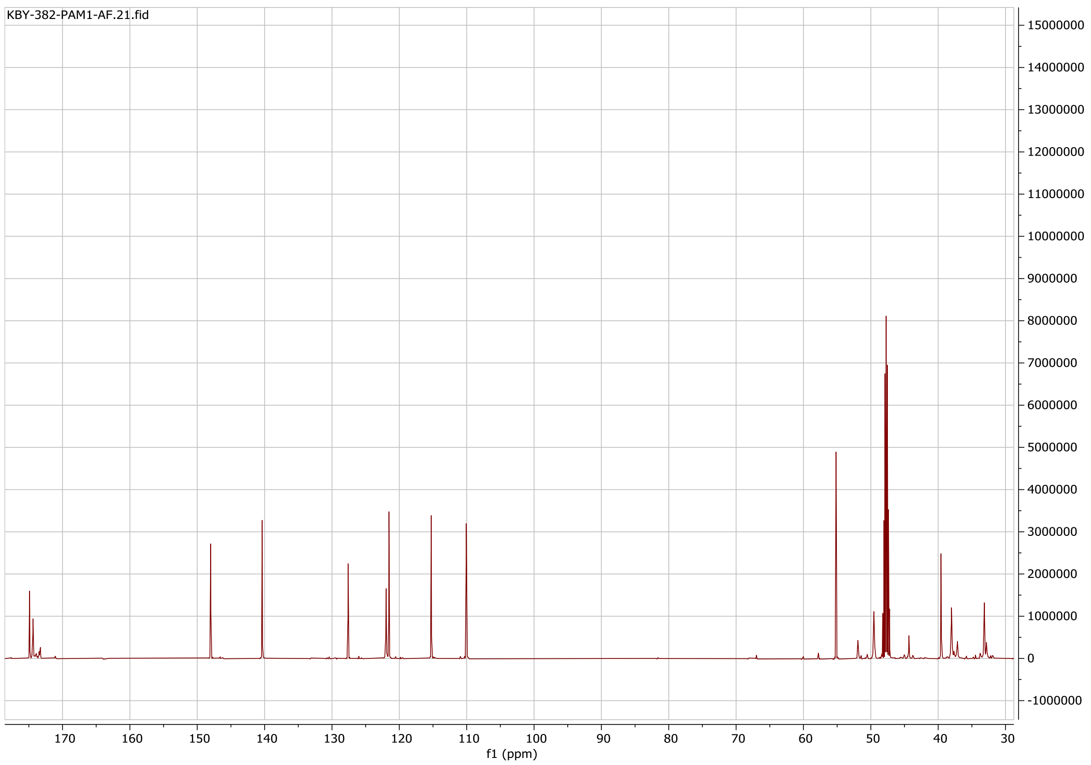

Figure S39: ^{1}H NMR of PAMAM-2-FA in CD_3OD

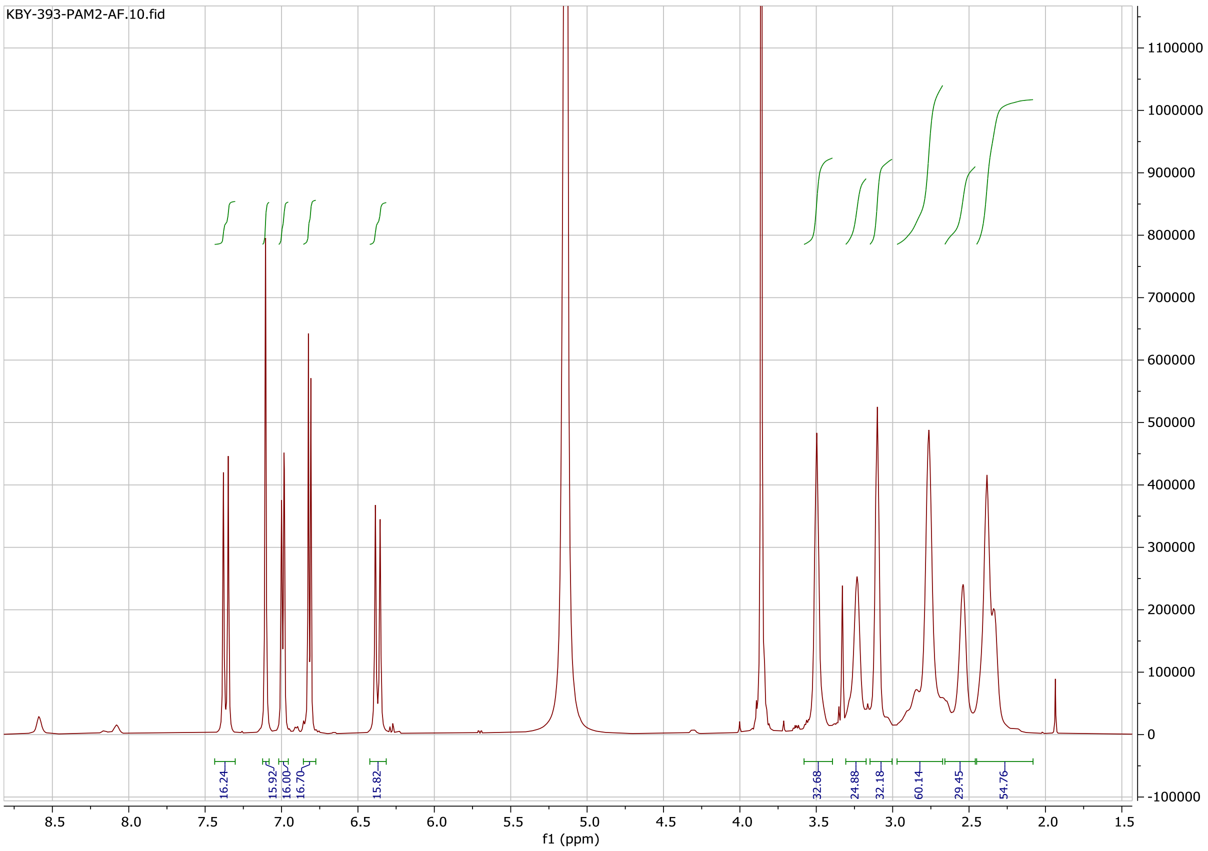

Figure S40: IR of PAMAM-2-FA

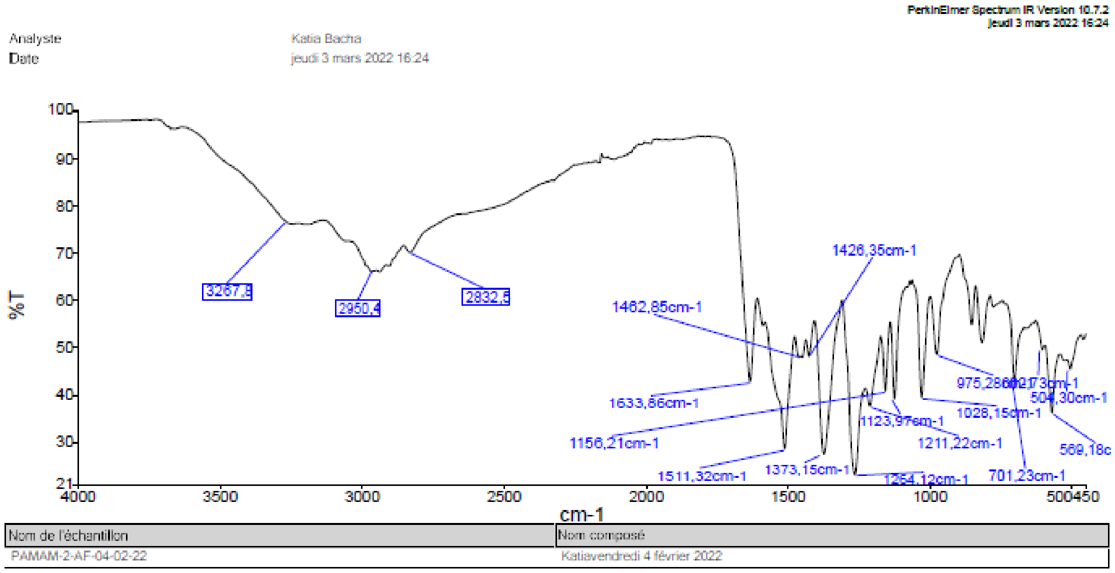

Figure S41: ^{1}H NMR of PAMAM-1-CA in $(CD_3)_2SO$

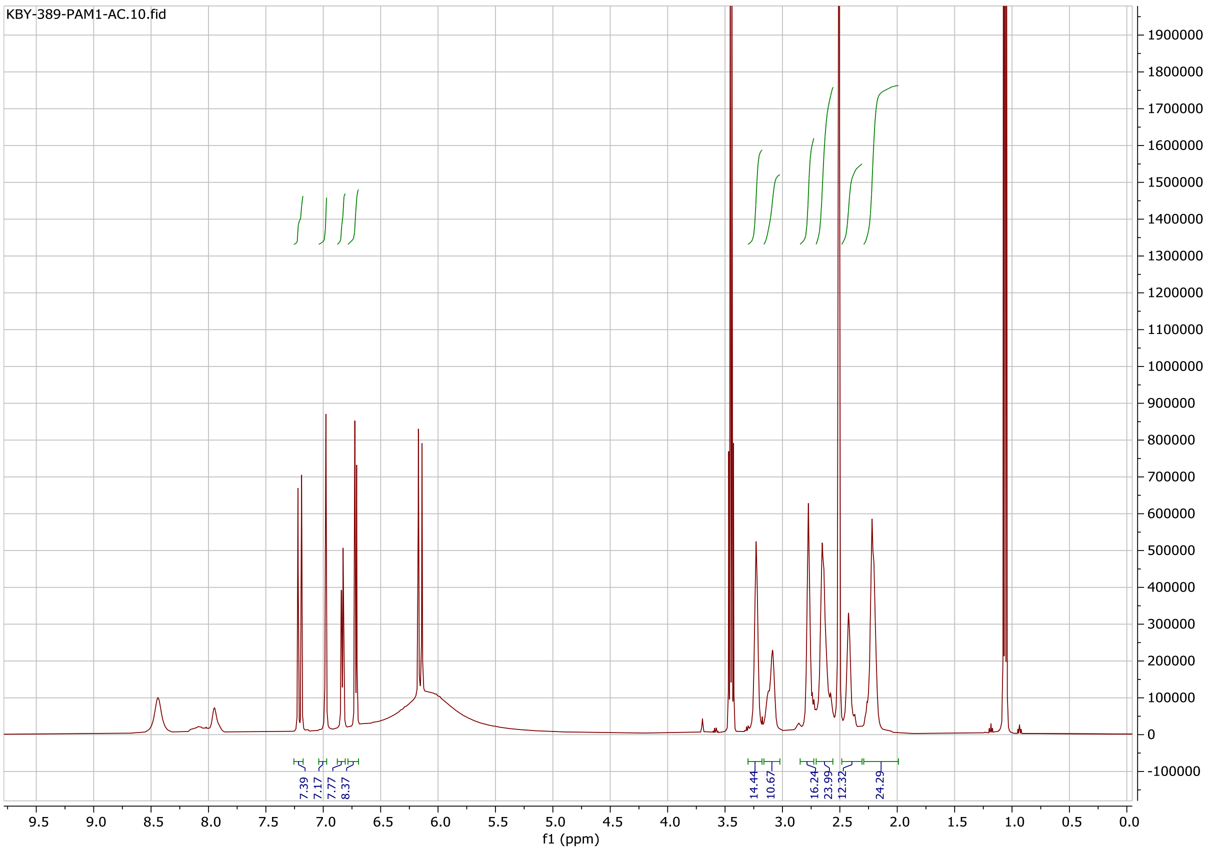

Figure S42: ^{13}C NMR of PAMAM-1-CA in $(CD_3)_2SO$

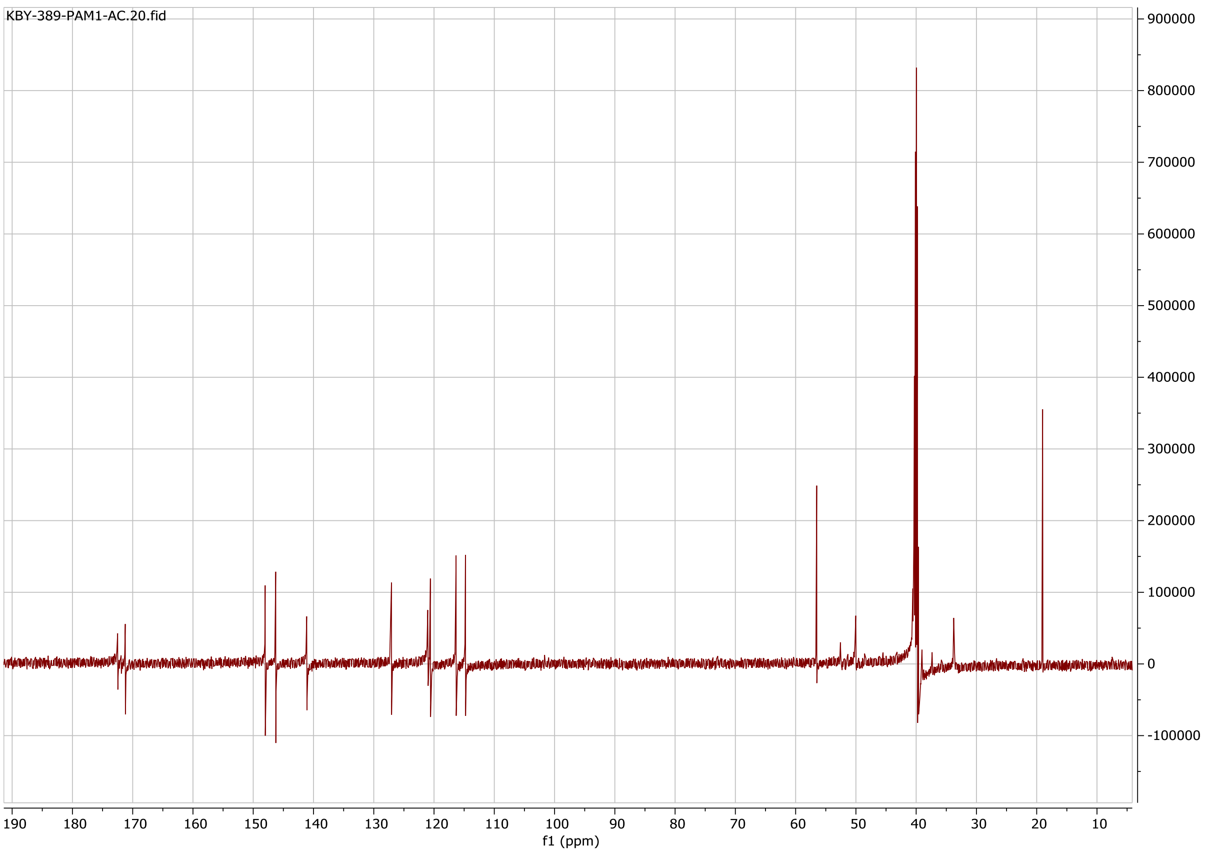

Figure S43: IR of PAMAM-1-CA

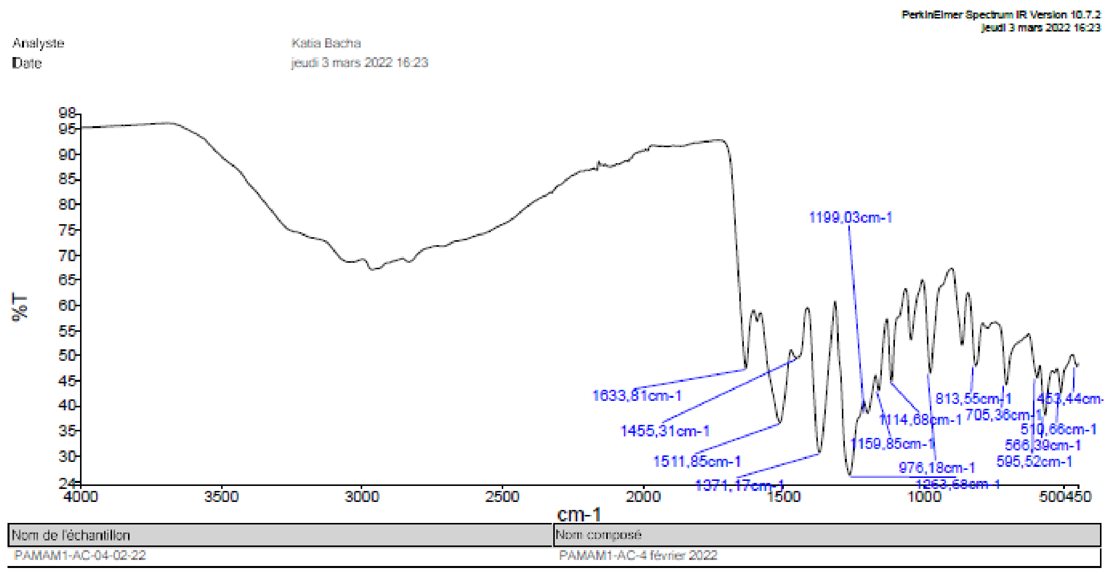

Figure S44: ^1H NMR of PAMAM-2-CA in $(CD_3)_2SO$

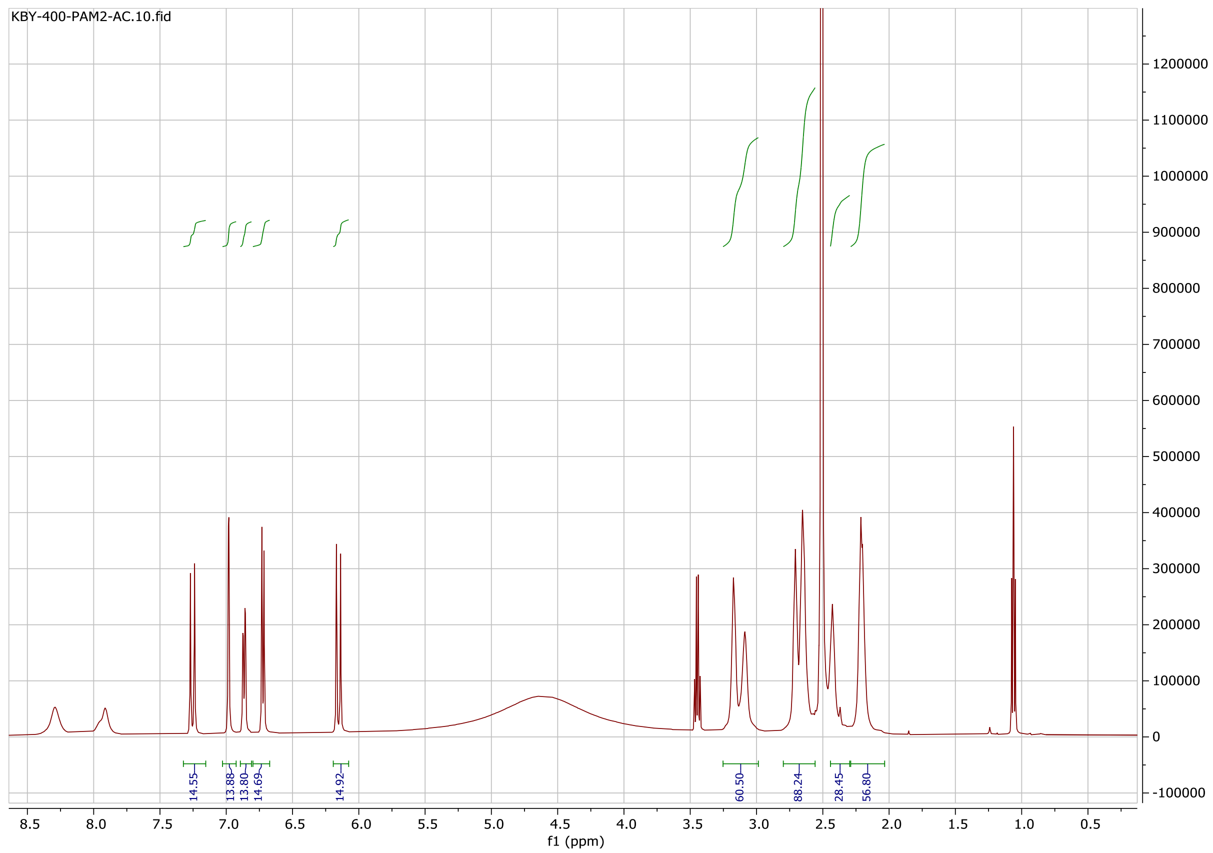

Figure S45: IR of PAMAM-2-CA

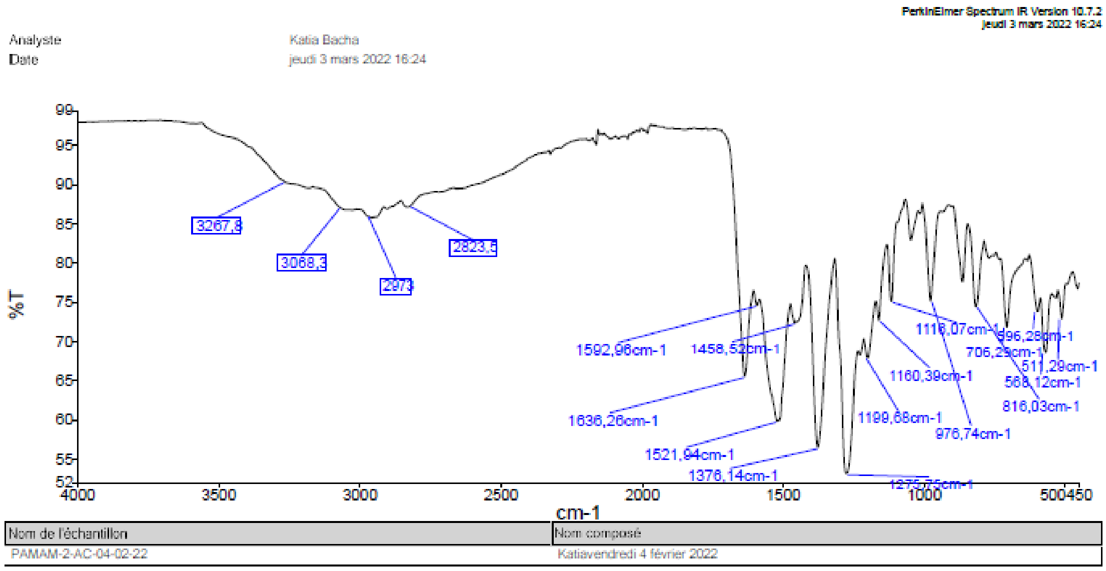

Figure S46: ^{1}H NMR of PAMAM-3-CA in $(CD_3)_2SO$

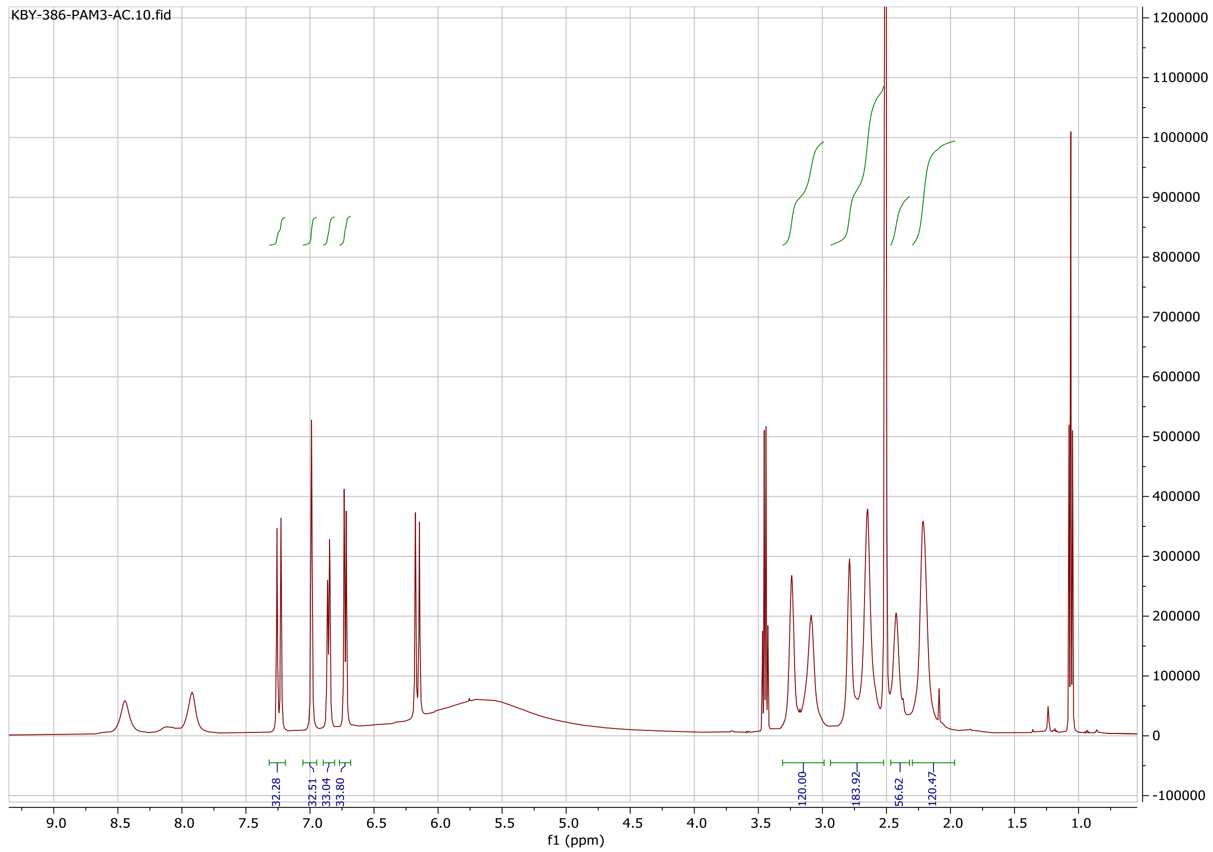

Figure S47: IR of PAMAM-3-CA

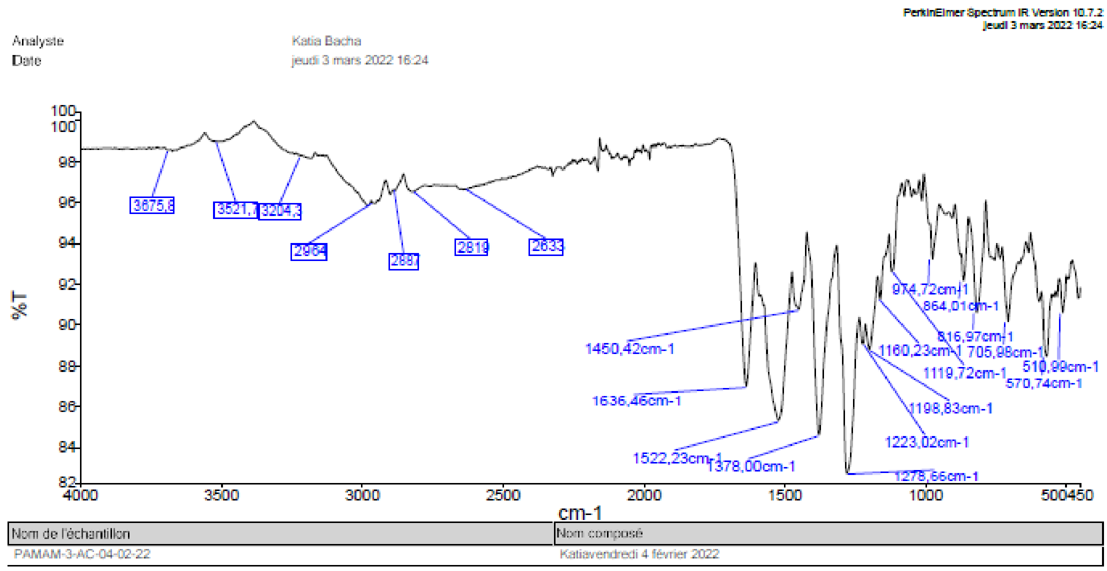

IR of PAMAM-3-CA

Figure S48: Inhibition of DPPH (300µmol or 118.3mg/L) by caffeic acid derived-PAMAM dendrimers

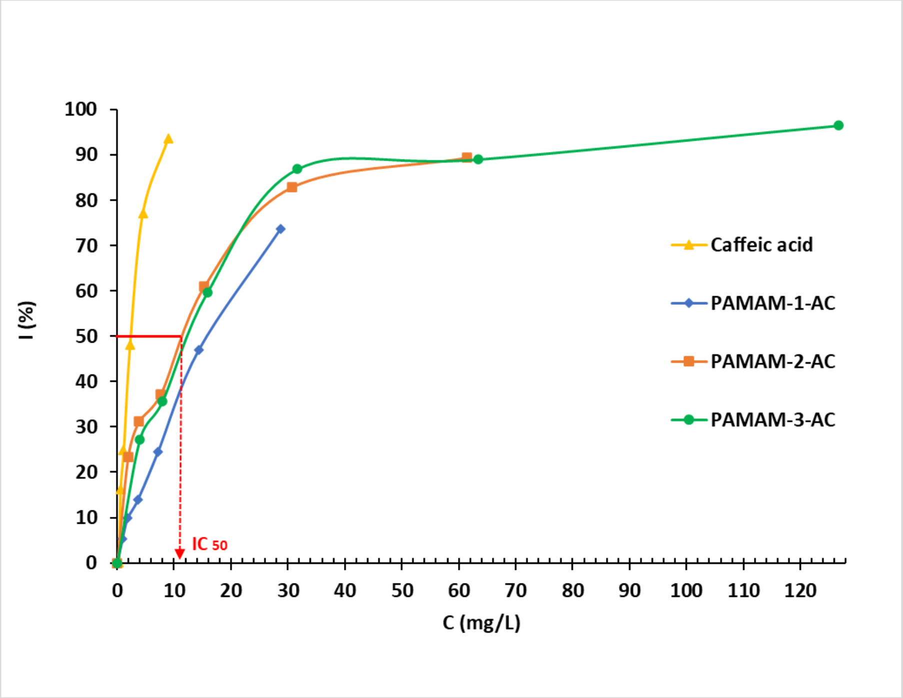

Figure S49: Inhibition of DPPH (300µmol or 118.3mg/L) by ionic antioxidant dendrimers

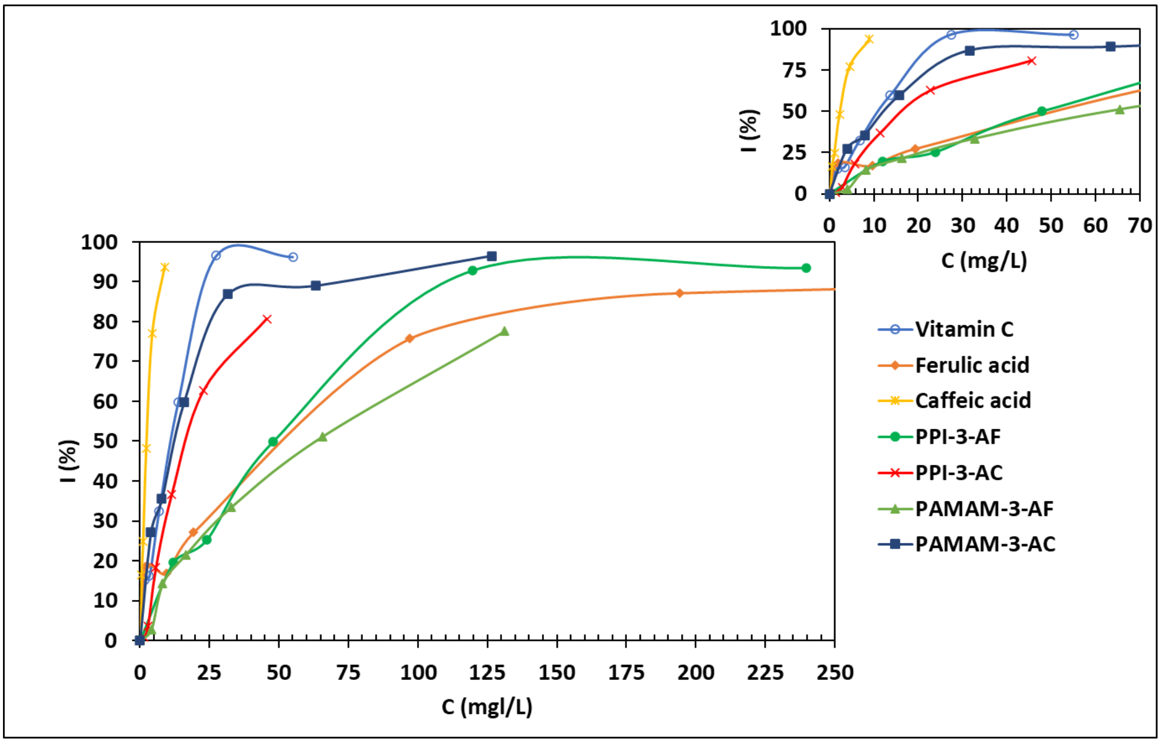

Table S1: IC_{50} (mg/L)

Entry	Samples	IC_{50} (mg/L)	Standard deviation
1	Vitamin C	10.92	± 0.3
2	Ferulic acid	52.42	± 4.6
3	PPI-1-FA (4 FA units)	32.79	± 1.9
4	PPI-2-FA (8 FA units)	39.61	± 4.9
5	PPI-3-FA (16 FA units)	47.92	± 12.4
6	PAMAM-1-FA (8 FA units)	65.63	± 5.9
7	PAMAM-2-FA (16 FA units)	54.72	± 8.3
8	PAMAM-3-FA (32 FA units)	62.99	± 10.5
9	Caffeic acid	2.34	± 0.2
10	PPI-1-CA (4 CA units)	10.47	± 1.3
11	PPI-2-CA (8 CA units)	16.64	± 2.2
12	PPI-3-CA (16 CA units)	15.99	± 5.0
13	PAMAM-1-CA (8 CA units)	15.50	± 3.1
14	PAMAM-2-CA (16 CA units)	11.04	± 5.5
15	PAMAM-3-CA (32 CA units)	12.67	± 5.1
16	PAMAM-3-PhA	5868.44	± 52.6
